# Vitamin A Deficiency and Alterations in the Extracellular Matrix

**DOI:** 10.3390/nu6114984

**Published:** 2014-11-10

**Authors:** Teresa Barber, Guillermo Esteban-Pretel, María Pilar Marín, Joaquín Timoneda

**Affiliations:** 1 Departamento de Bioquímica y Biología Molecular, Facultad de Farmacia, Universitat de Valencia, Avda V. Andrés Estellés s/n, 46100-Burjassot, Spain; E-Mails: guiespre@alumni.uv.es (G.E.-P.); joaquin.timoneda@uv.es (J.T.); 2 Unidad de Microscopía IIS La Fe Valencia, Avda Campanar, 21, 46009-Valencia, Spain; E-Mail: marin_marmue@gva.es

**Keywords:** vitamin A deficiency, retinoic acid, extracellular matrix, basement membrane, collagen, kidney, lung, liver

## Abstract

Vitamin A or retinol which is the natural precursor of several biologically active metabolites can be considered the most multifunctional vitamin in mammals. Its deficiency is currently, along with protein malnutrition, the most serious and common nutritional disorder worldwide. It is necessary for normal embryonic development and postnatal tissue homeostasis, and exerts important effects on cell proliferation, differentiation and apoptosis. These actions are produced mainly by regulating the expression of a variety of proteins through transcriptional and non-transcriptional mechanisms. Extracellular matrix proteins are among those whose synthesis is known to be modulated by vitamin A. Retinoic acid, the main biologically active form of vitamin A, influences the expression of collagens, laminins, entactin, fibronectin, elastin and proteoglycans, which are the major components of the extracellular matrix. Consequently, the structure and macromolecular composition of this extracellular compartment is profoundly altered as a result of vitamin A deficiency. As cell behavior, differentiation and apoptosis, and tissue mechanics are influenced by the extracellular matrix, its modifications potentially compromise organ function and may lead to disease. This review focuses on the effects of lack of vitamin A in the extracellular matrix of several organs and discusses possible molecular mechanisms and pathologic implications.

## 1. Introduction

Vitamin A specifically refers to all-*trans* retinol but is also used as a generic term for isoprenoid compounds that qualitatively exhibit the biological activities of all-*trans* retinol [1]. It is converted successively by two oxidative reactions into its biologically active derivatives, retinaldehyde and retinoic acid (RA), which can exist as all-*trans*- and several *cis*-isomers. These compounds, together with all the natural and synthetic compounds with vitamin A activity, are collectively known as retinoids. Biologically-occurring retinoids are necessary for normal embryonic development and organogenesis, and exert major effects on postnatal tissue homeostasis, vision, reproduction, immune function, growth, cellular differentiation, proliferation and apoptosis [2,3,4,5,6,7,8]. Most of these functions of retinoids are dependent on RA interactions with two types of transcription factors, retinoic acid receptors (RARs) and retinoid X receptors (RXRs), each with three subtypes (α, β and γ) and several isoforms. They regulate the expression of multiple genes by acting as heterodimers (RAR/RXR) or homodimers (RXR/RXR). All-*trans*-RA activates RARs, while 9-*cis*-RA serves as a pan-agonist for RARs and RXRs [9]. Upon activation by RA, the dimerized receptor binds to the retinoic acid response element (RARE) in the promoter region of target genes and stimulates their transcription, usually by recruiting other transcriptional co-activator proteins. In the absence of the agonist, DNA-bound receptors can recruit co-repressor proteins and act as transcriptional repressors [8,10]. Recently transcriptional effects for retinol and retinal have also been described [11,12].

In agreement with the multiple functions of retinoids, deficiency of vitamin A (VAD) leads to a spectrum of clinical manifestations, known as vitamin A deficiency disorders (VADD). These include mild to severe xerophtalmia, squamous metaplasia of transitional and glandular epithelia, growth disturbances, anemia, susceptibility to infections and increased mortality. In laboratory animals, VAD during gestation results in fetuses with congenital malformations that affect ocular, cardiac, respiratory and urogenital systems, or even fetal death if deficiency is severe enough [13,14].

The extracellular matrix (ECM) constitutes the non-cellular compartment within any tissue and organ, and provides not only a scaffold for cell attachment, but also information for adequate cell behavior and tissue function. It is formed by a complex array of highly cross-linked macromolecules, such as collagens, laminins, fibrilins, elastins, fibronectins and several proteoglycans [15,16]. The differential distribution of these proteins results in structurally and biochemically heterogeneous and tissue-specific ECMs. A reciprocal interaction occurs between cells and ECM. On the one hand, ECM molecules are synthesized and assembled by the cells residing within it and, on the other, cells receive information from the ECM components they contact. Cells interact with the ECM through ECM receptors, including the syndecans, a four-member group of transmembrane cell surface proteoglycans, dystroglycan, a heterodimer component of muscle dystrophin-glycoprotein complex, discoidin domain receptors, a two-member family of tyrosine kinase receptors activated by native collagen, and heterodimeric integrins, which constitute the major class of ECM receptors. These specific cell-ECM interactions, performed independently or synergistically with other growth factor receptors, trigger the intracellular signaling pathways that regulate cell processes, such as migration, proliferation, differentiation, metabolism and apoptosis [15,16,17,18,19]. Both the composition of the ECM and the repertoire of ECM receptors determine the responses of cells. Consequently, changes in the structure or composition of the ECM may potentially alter cell and organ responses, and lead to the development or progression of disease [20].

Among the proteins whose synthesis is regulated by retinoids we find those of the ECM. In addition to modulating the synthesis of many ECM proteins, such as collagens, laminins, fibronectin and elastin, RA also affects the expression of their cell membrane receptors. Therefore, the structure and composition of the ECM is modified in VAD, and this altered ECM can act as a contributory factor in the deleterious effects of vitamin deficiency. This review focuses on the effects of lack of vitamin A on the ECM of several organs, and discusses possible mechanisms and pathologic implications.

## 2. Extracellular Matrix

**Figure 1:**
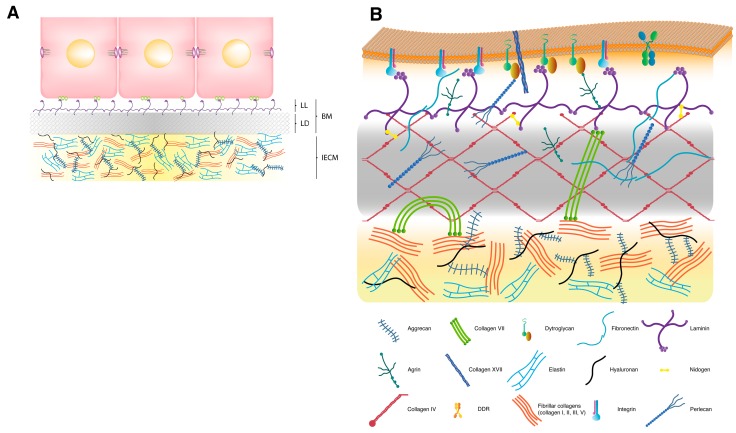
Schematic model of the extracellular matrix and the basement membrane. (**A**) Extracellular matrix (ECM). In many tissues, such as glandular, stratified and certain complex epithelia, the ECM is formed by a basement membrane (BM), which is connected to the epithelial cells, and an interstitial extracellular matrix (IECM), which constitutes most ECM space. Cells adopt basal-apical polarity and interact with BM components through specific cell surface receptors located on their basal side. As in transmission electron microscopy after conventional fixation, the BM is composed of two layers: (1) the lamina lucida (LL), an electron-lucent layer that lies immediately adjacent to the cell; and (2) the lamina densa (LD), an electron-dense layer that comes into contact with the IECM. The lamina lucida is not detectable in electron microscopy specimens fixed by the milder method of freeze substitution. (**B**) Basement membrane. The basic structure of a BM is formed by two independent networks of laminin and collagen IV, which are non-covalently interconnected by nidogen and perlecan. The self-assembled collagen IV network is recruited together with other BM components, including nidogen, perlecan and agrin, to the nascent laminin scaffold. Agrin and perlecan provide additional connections between the BM and the cell surface. Native collagens also have the possibility of contacting cells through plasma membrane receptors, such as DDRs (not shown in the figure). Several molecules, such as collagens VII, XV and XVIII, are known to act as linkers between the BM and the adjacent IECM. For clarity purposes, only collagen VII aggregates, which may interact with fibronectin, laminin, collagen I and collagen IV, and form the anchoring fibrils, are included in the figure.

The ECM is a complex array of several distinct families of molecules which occupy the non-cellular compartment in any tissue and organ outside blood. In addition to providing tissues with structural strength and flexibility, and cells with anchoring support, the ECM performs important signaling functions which modulate migration, proliferation, differentiation, metabolism and survival of contacting cells [20]. It exists as an interstitial ECM, which constitutes most of the extracellular mass in the majority of tissues and confers them with a definite architecture and integrity, and as a basement membrane (BM), a sheet-like specialized form that underlies epithelia and endothelia, and surrounds certain other tissues and cell types, such as neurons, muscle fibers and adipocytes, separating them from and connecting them to the adjacent interstitial matrix (Figure 1A) [21]. In the special cases of renal glomerulus, pulmonary alveolus, muscle, and certain parts of the central nervous system, the IECM has been eliminated by merging the BMs of two juxtaposed cellular layers.

### 2.1. Interstitial Extracellular Matrix

Almost 300 proteins have been defined as constituents of the core matrisome [16]. However, several groups of molecules which include the collagens, elastins, fibrilins, laminins, hyaluronan and some proteoglycans are considered the major components of ECMs (Figure 1A). Fibril-forming proteins, such as collagens I, II, III and V, elastins, and fibrillins, are characteristic components of the interstitial ECM. Collagens are triple-helical molecules with collagenous domains of varying lengths which result from the association of subunits, called α-chains, as homotrimers or as restricted sets of heterotrimers. In the case of fibril-forming collagens, these trimeric, rod-like molecules assemble into fibrils and are stabilized by tissue-specific crosslinking reactions that confer further resistance to shear stress [22]. Collagens I, III and V appear in fibrils of most tissues, whereas collagen II predominates in the cartilage and vitreous humor. Although several types of collagen generally associate to form fibrils, usually one type predominates. These collagen fibers coexist with the elastic fibers that provide elastic recoil to tissues, and are especially abundant in blood vessels, lung and skin. Elastin is the predominant protein comprising approximately 90% of mature elastic fiber and presents isoform diversity that results from the tissue-specific alternative splicing of a single primary transcript. It is secreted as a monomer which self-associates and cross-links extracellularly to form microaggregates, a process probably guided by fibronectin, integrins and heparan sulphate proteoglycans. Elastin microaggregates are transferred to a microfibril scaffold of fibrillin molecules where they coalesce and are further stabilized by additional cross-links to form the insoluble core of elastic fibers [23]. Interspersed between or bound to collagen and elastic fibers are found the proteoglycans, such as aggrecan, which, in addition to their tissue hydrating properties, regulate collagen fibrillogenesis and growth factor signaling [24]. Another fibril-forming and ubiquitous ECM component is fibronectin, a multidomain glycoprotein that is synthesized by many cell types and secreted as a dimer of antiparallel subunits linked covalently by two disulfide bonds at their *C*-termini [25,26,27]. It can be found in plasma as a soluble dimer, synthesized mainly by hepatocytes, and in the ECM to form insoluble fibrilar multimers after being secreted by fibroblasts and many other cell types. This fibronectin fibril assembly is a cell-dependent process initiated by the binding of secreted dimers to transmembrane integrin receptors, which transforms the fibronectin dimer from a compact into an extended conformation. This extension exposes the binding sites required for fibronectin incorporation into fibrils and for other intermolecular interactions [28], such as with collagen, preferentially in its denatured form.

### 2.2. Basement Membrane

Major components of BMs are the net-forming macromolecules collagen IV and laminins, the heparan sulfate proteoglycans perlecan and/or agrin, and the nidogens (Figure 1B) [29]. Collagen IV is a group of trimeric molecules whose subunit composition differs. In mammals, six homologous, but genetically different α-chains (α1–6), can assemble to form the heterotrimeric molecule. Yet, although many potential combinations are possible, the six collagen IV α-chains apparently form only three sets of triple-helical molecules called protomers (α1, α1, α2; α3, α4, α5; and α5, α5, α6) [30,31]. Each protomer has three domains: a short triple helical domain rich in cysteine and lysine at the *N*-terminus, called the 7S domain because of its sedimentation coefficient; a long, triple helical collagenous domain in the middle of the molecule; and a noncollagenous (NC1) trimer at the *C*-terminus. Unlike fibrillar collagens, numerous short interruptions in the collagenous sequence ensure enough flexibility to loop and supercoil protomers into networks, and provide sites for cell binding and interchain cross-linking [32]. The specificity for chain selection to form the protomer lies in each chain’s NC1 domain [33,34,35,36]. Once in the extracellular space, secreted protomers self-associate to form networks through the interactions between the 7S domains of four protomers, the NC1 domains of two protomers, and the lateral associations of collagenous domains. This network provides a scaffold for the attachment of cells and other ECM molecules, such as laminin, perlecan and nidogen. Laminin is the most abundant noncollagenous protein in BMs. It is a large hetrotrimeric glycoprotein comprised of one each of five α, four β and three γ chains joined through a long coiled-coil domain. As with collagen IV, there are many potential combinations but only 16 isoforms have been confirmed in vertebrates [37]. As deduced from rotary shadowing electron microscopy, laminin is a cruciform molecule with the long arm of the cross formed by an α-helical coiled coil of the three chains, and the three short arms by one chain each. According to the three-arm model, secreted laminin molecules self-assemble into a honeycomb-like polymer with the ternary nodes resulting from the calcium-dependent interactions between the *N*-terminal domains of three short arms of different laminin monomers (Figure 1B) [38,39]. This polymerization process is promoted and organized by laminin recruitment at the plasma membrane of BM-associated cells predominantly via direct interactions of laminin LG domains with cell surface receptors, such as integrins, α-dystroglycan and heparan sulfates. Laminins are also indirectly connected to the cell surface through BM proteoglycans. Perlecan and agrin, the major proteoglycans in BMs, are multifunctional heparan sulfate proteoglycans formed by a multimodular protein core to which several heparan sulfate chains are attached, three at the central region of agrin and three at the *N*-terminal domain of perlecan. Many of these protein modules share a sequential, structural and functional homology with ECM proteins, growth factors and surface receptors [40], and provide interaction sites with other ECM components and cells. The *N*-terminal domain of agrin binds to laminin and its *C*-terminal LG domains bind to α-dystroglycan. Perlecan, in turn, may simultaneously interact with laminin or collagen IV through its *N*-terminal heparin sulfate chains and with α-dystroglycan through its *C*-terminal LG domains (Figure 1B). In addition to accomplish these interactions, heparan sulfate moieties also have the potential to bind and modulate the function of growth factors, such as fibroblast growth factor, transforming growth factor-β (TGF-β), vascular endothelial growth factor and platelet derived growth factor, and numerous cytokines and chemokines [41,42]. Nidogens, also known as entactins, are two virtually ubiquitous glycoproteins in BMs. They are encoded by two different genes and, in spite of low sequence homology, they share a highly similar structural organization. Both nidogens have a protein core composed of three globular domains separated by two rod-like segments, and both show a similar repertoire of binding partners, particularly collagen IV, laminin, perlecan and fibronectin [43].

Therefore, based on available data, BMs can be considered to be constituted by two independent intertwined networks of polymeric laminin and type IV collagen which are bridged by non-covalent interactions with nidogens and/or heparan sulfate proteoglycans [38,44,45].

### 2.3. Transmembrane Extracellular Matrix Receptors

**Figure 2:**
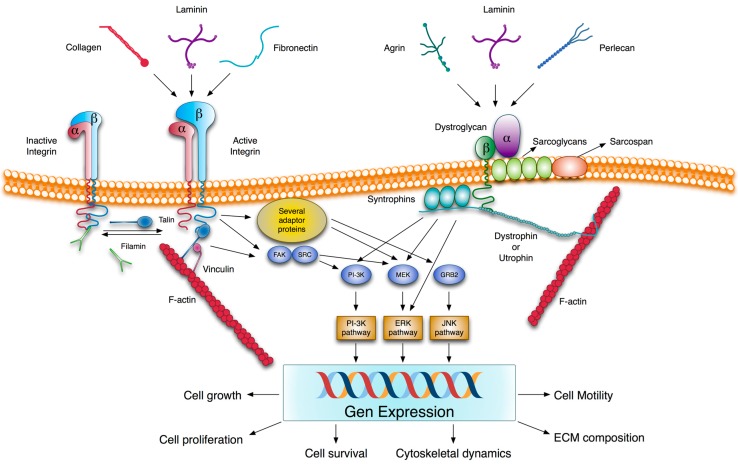
Integrin- and dystroglycan-mediated ECM signaling. Several ECM components are able to interact with integrins and dystroglycans. Unlike dystroglycan, integrins are present on the cell surface as inactive and active forms. The conformational switch from low to high ligand affinity may be regulated by different ways. These include β-subunit cytoplasmic tail phosphorylation and competitive binding to this β-subunit tail between activators and inhibitors, such as cytoskeletal proteins talin and filamin, respectively. Ligand-integrin binding induces the recruitment of several adaptor proteins and the phosphorylation of some protein kinases such as focal adhesion kinase (FAK) which trigger different signaling pathways. In addition, integrins are linked to actin filaments through proteins, such as talin, filamin or actinin. Similarly, the interaction of α-dystroglycan with the LG domain containing ligands transduces extracellular information via β-dystroglycan to generate intracellular signals, which are mediated by signaling pathways, also activated by integrins. In fact, there is much evidence to suggest a cross-talk between both receptors. The signaling pathways induced by integrins and dystroglycans modulate gene expression patterns and impact cell behavior. The molecular organization of both receptors with their associated proteins provides a physical connection between the ECM and the cytoskeleton, and a way to transmit mechanical cues between them. ERK, extracellular signal-regulated kinase; FAK, focal adhesion kinase; GRB2, growth factor receptor-bound protein 2; JNK, c-Jun *N*-terminal kinase; MEK, MAPK kinase; PI-3K, phosphatidylinositol 3-kinase; Src, protein tyrosine kinase encoded by the c-src gene.

As mentioned above, all the ECM components are multi-modular molecules that are capable of numerous potential interactions with themselves and with cells. Accordingly, the attachment of cells to the ECM is mediated by interactions of cell surface receptors with virtually any of its components (Figure 2). Yet in spite of interaction promiscuity, there is some degree of ligand specificity which depends on the subunit composition of both the ligand and receptor. Several types of receptors are involved in these interactions, and the integrin family is the major one. Integrins are the heterodimeric transmembrane receptors that result from non-covalent combinations of eighteen α and eight β subunits to form 24 αβ heterodimers with different, but overlapping, binding specificities and different tissue distribution. Each subunit has a large extracellular region with several domains, a single membrane-spanning helix and, usually, a short unstructured cytoplasmic tail devoid of enzymatic features [46]. Many integrin receptors recognize short specific sequences such as RGD, LDV, collagenous GFOGER or some other related ones, and, consequently, they are able to bind a wide variety of ECM proteins [47]. Upon activation by ligand signaling or mechanical forces, integrin cytoplasmic tails recruit a wide array of factors, including signaling and adaptor proteins, which constitute the cell-matrix adhesion complexes. These complexes mediate signaling processes from the ECM to the cell that control cell differentiation, proliferation, survival and migration [48,49]. Integrins are bidirectional signal transmitters; not only do they transmit information from extracellular stimuli to induce intracellular changes, but intracellular stimuli can also result in extracellular modifications such as the activation of integrins themselves. This activation facilitates cell-ECM interaction, integrin clustering, and the formation of stable cell-matrix adhesion complexes [50].

Akin to the integrin receptors is the dystroglycan, another transmembrane heterodimer of α- and β-subunits, which also links ECM glycoproteins to the intracellular cytoskeletal network. Both subunits are translated from a single transcript and are subject to extensive co- and post-translational modifications, including proteolysis, phosphorylation and glycosylation. Dystroglycan is a receptor for several BM proteins, principally those containing laminin LG domains such as laminin itself, perlecan and agrin. The α-dystroglycan, located on the outer surface of the plasma membrane, binds to BM proteins and β-dystroglycan, a transmembrane component of the complex, interacts extracellularly with α-dystroglycan and intracellularly with dystrophin, or its autosomal homolog utrophin, which, in turn, has binding affinity with F-actin. The β-subunit cytodomain and dystrophin/utrophin are associated with other integral (sarcoglycans and sarcospan) and peripheral (syntrophins and dystrobrevins) membrane proteins, which are involved in signal transduction (Figure 2) [51]. Dystroglycan and its associated proteins constitute the dystrophin-glycoprotein complex which has beensuggested to perform an important mechanical function in stabilizing the muscle plasma membrane and in regulating signal transduction pathways [52].

A third type of ECM receptors, widely expressed in embryonic and adult tissues, is homologous discoidin domain receptors DDR1 and DDR2, which display a tyrosine kinase activity in their cytoplasmic domains. They constitute a subfamily of receptor tyrosine kinases and are unique among these receptors in that they are activated by an ECM component [53,54]. Both recognize native forms of collagen as their only known activating ligands, but with slightly different binding specificities. They both bind to fibrillar collagens, such as collagens I–III and V, but only DDR1 is activated by non-fibrillar BM collagen IV, while collagen glycosylation is important only for DDR2 stimulation [55,56]. Upon collagen binding, DDRs undergo a slow, sustained tyrosine autophosphorylation within their intracellular domains, which generates docking sites for the SH2, SH3 and PTB domain-containing proteins. The formation of these protein complexes may trigger the activation of specific signaling pathways, which may synergize or antagonize the responses induced by other ECM-activated receptors, such as integrins [57,58]. Moreover, DDRs may act in concert with other signaling receptors, including the Wnt5a-Frizzled [59] and Notch1 [60] receptors and the insulin receptor [61] for DDR1 and DDR2, respectively [62].

Native collagen is also a ligand for two other related and cell type-restricted expression receptors, glycoprotein VI (GPVI) and leukocyte-associated immunoglobulin-like receptor I (LAIR-1). Both belong to the Ig superfamily of surface receptors, are encoded in the leukocyte receptor cluster on human chromosome 19q13.4 and bind to Gly-Pro-Hyp collagen repeats [63,64]. However, GPVI is an activating receptor whose expression is restricted to megakaryocytes and platelets, and LAIR-1 is an inhibitory receptor expressed in almost all immune cells [65,66].

Through these transmembrane receptors, the ECM not only acts as a scaffold for cell attachment but also signals to cells, and contributes to control cell behavior, polarity, and differentiation.

## 3. Retinoid Signaling

**Figure 3:**
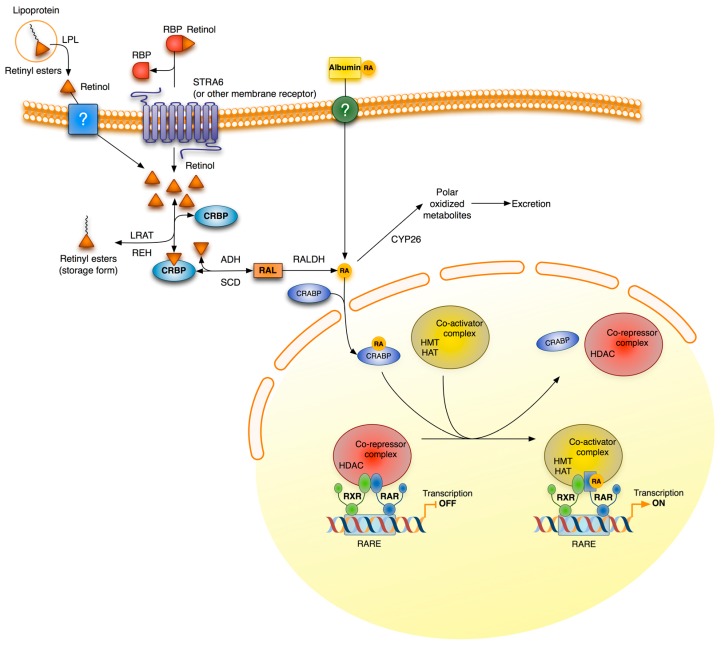
General scheme of retinoic acid signaling and metabolism. Retinoids enter cells via several possible routes. In extrahepatic tissues, RBP-bound retinol enters cells through the STRA6 receptor or any other related receptor; and free retinol, derived from lipoproteins by the action of lipoprotein lipase, and albumin-bound RA, which is present in small amounts in plasma, may enter by passive diffusion and/or by any uncharacterized receptor. Hepatocytes and, to a lesser extent, also extrahepatic cells, obtain retinyl esters by lipoprotein uptake. Inside cells, retinol is esterified and stored as retinyl esters or is metabolized to retinoic acid (RA) by two sequential oxidations. It is thought that intracellular retinoid-binding proteins, such as cellular retinol-binding proteins (CRBP) and cellular retinoic acid-binding proteins (CRABP), participate in the coordination of these processes. RA is degraded to more polar, less bio-active metabolites by enzymes of the CYP26 family. CRABP-bound RA is translocated to the nucleus where it binds to the retinoic acid receptor (RAR) and initiates gene transcription. This effect is produced even in the absence of an RXR agonist, however the binding of agonists to both receptor partners improves transcription efficiency. ADH, alcohol dehydrogenases; CRBP, cellular retinol-binding protein; CRABP, cellular retinoic acid binding protein; CYP26, family 26 of cytochrome P450 enzymes; HAT, histone acetyltransferase; HDAC, histone deacetylase; HMT, histone methyltransferase; LPL, lipoprotein lipase; LRAT, lecithin retinol acyltransferase; RALDH, retinaldehyde dehydrogenase; RAR, retinoic acid receptor; RARE, retinoic acid response element; RBP, retinol binding protein; REH, retinyl ester hydrolases; RXR, retinoid X receptor; SDR, short-chain dehydrogenase/reductases.

Retinoids are compounds that are functionally related to vitamin A, including its natural derivatives and synthetic analogs. Retinol, retinal and RA, which differ in terms of the oxidation state of their polar group, are the physiologically important natural retinoids. Inside cells, retinol is oxidized reversibly to retinal by cytosolic alcohol dehydrogenases and microsomal short-chain dehydrogenases, while retinal can be further irreversibly oxidized to RA by retinaldehyde dehydrogenase. RA, the main active derivative of vitamin A, regulates gene transcription by binding to nuclear receptors, RARs and retinoid X receptors RXRs (Figure 3). Both receptors have three subtypes (α, β, and γ) each with different isoforms which result from differential promoter usage and splicing. RARs require heterodimerization with RXRs for effective DNA binding and function. However, RXRs can form homodimers, or even heterodimers, with other non-RA associated nuclear receptors to mediate alternative signaling pathways. All-trans RA, the predominant isomer *in vivo*, and 9-*cis* RA activate RARs, whereas RXRs are activated only by 9-*cis* RA. Despite their lower affinity, all-trans RA can also activate peroxisome proliferation-activated receptor β/δ (PPAR β/δ), a nuclear receptor involved in energy balance, lipid metabolism and insulin resistance, and reveal a role for RA in these processes [67]. RAR-RXR receptors bind to specific sequences on DNA, the RARE, whose consensus sequence is a two direct repeat of the hexameric core (A/G)G(G/T)TCA, but separated by a several base pair spacer. In the absence of a ligand, DNA-bound RARα associates with corepressor proteins, which recruit protein complexes with histone deacetylase activity. By deacetylating lysine residues of histones, these complexes maintain chromatin in a condensed, repressed state. Upon ligand binding, RARα undergoes conformational modifications, which allow the exchange of coactivators for corepressors (Figure 3). Coactivators recruit concertedly protein complexes with several enzymatic activities, such as histone acetyltransferases, histone methyltransferases, DNA-dependent ATPases, chromatin remodelers, general transcription factors and RNA polymerase II, to result in the increased transcription of target genes. RA also increases the binding of receptors to DNA by a yet incompletely known mechanism, although the phosphorylation of specific and conserved serine residues appears to be involved [68,69,70].

In addition to these transcriptional actions, retinoids also exert non-genomic effects. These do not involve direct gene transcription, but the ligand-induced modulation of signal transduction pathways by nuclear retinoic acid receptors. Indeed, it has been shown in different cell types that RA activates several kinase cascades rapidly and transiently. So RA causes a rapid phosphorylation of transcription factor CREB (cAMP response element binding protein) in neuronal cells, leading to a stimulation of the transcription of CREB-dependent genes, such as c-fos, whose promoters do not contain RAREs. CREB is a substrate for extracellular signal-regulated kinase 1/2 (ERK1/2) and it has also been shown that RA, through RARs, activates the PI3K and ERK1/2 MAPK signaling pathways in neuroblastoma cells [71,72]. In addition, a rapid increase in adenylate cyclase activity and the intracellular cAMP level has been observed in human leukemia cells after treatment with RA. This cAMP induction is accompanied by the up-regulation of protein kinase A activity, RARα phosphorylation and RARα transcriptional activity [73]. Treatment of these cells with RA also induces the activation of protein kinase Cδ and its association with RARα leading to an increased transcription of RARE-dependent genes [74]. Other signaling pathways, such as TGF-β/Smad, also appear to be affected non-transcriptionally by RA. It has been proposed that by directly interacting with Smad3, RARs may function as either coactivators of the Smad pathway in the absence of agonists or inhibitors in its presence [75], and that RA activates a RARα-dependent phosphatase, which lowers the level of phospho-Smad2/3 induced by TGF-β [76].

**Figure 4:**
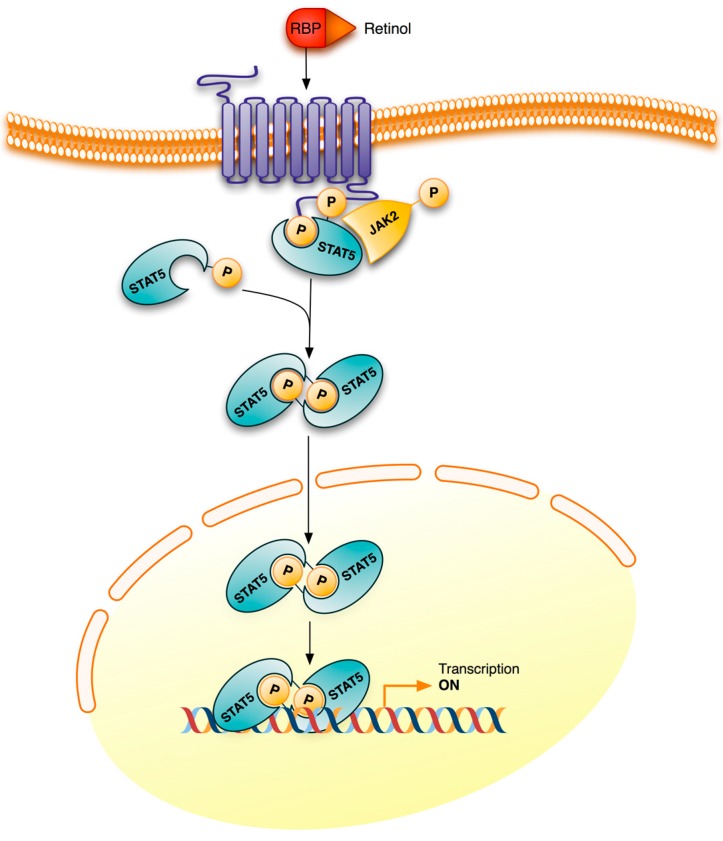
Retinol signaling. STRA6 is not only a plasma membrane transporter for retinol but also a signaling receptor. Binding of the retinol-RBP complex to the extracellular part of STRA6 triggers the phosphorylation of its cytosolic domain. The phosphorylated domain recruits and activates JAK2 which, in turn, phosphorylates STAT5, also bound to the phosphorylated receptor. Phosphorylated STAT5 dimers initiate transcription of their target genes. The letter P inside a circle denotes a phosphoryl group. JAK2, Janus kinase 2; RBP, retinol binding protein; STAT5, signal transducer and activator of transcription 5; STRA6, stimulated by retinoic acid 6.

Recently it has also been shown that retinol bound to RBP (retinol binding protein), its transport protein in plasma, activates intracellular signaling pathways (Figure 4). The binding of the retinol-RBP complex to STRA6 (stimulated by retinoic acid 6), a retinol plasma membrane receptor-transporter in some cells such as adipocytes, not only induces the transport of retinol into cells, but also triggers the JAK/STAT (Janus kinase/Signal Transducer and Activator of Transcription) signaling cascade. As a result, STAT target genes, such as SOCS3 (suppressor of cytokine signaling 3) which inhibits insulin signaling, and PPARγ which enhances lipid accumulation, are up-regulated [77]. Retinal, the natural metabolite of retinol oxidation, also modulates gene expression in differentiated adipocytes by inhibiting the activation of RXRs and PPARγ [11,78].

Through the RA-dependent regulation of gene expression and the RA- and retinol-induced activation of protein kinases, vitamin A signaling cross-talks with the transcriptional activity of other transcription factors and integrates its information with other signaling pathways. The existence of several natural retinoids with biological activity, the diversity of their action mechanisms and the variety of the participants involved are largely responsible for the pleiotropic effects of vitamin A.

## 4. Vitamin A Deficiency

### 4.1. Overview and Clinical Manifestations

The physiological plasma concentration of vitamin A is 1–2 µM and is under tight homeostatic control by the liver. According to the World Health Organization (WHO), a plasma retinol concentration below 0.70 µM (20 µg/dL) is indicative of VAD, while a concentration lower than 0.35 µM is indicative of severe deficiency. VAD has a wide range of undesirable clinical effects, which appear when liver reserves are exhausted. When liver vitamin A reserves fall below a critical concentration, thought to be approximately 20 µg/g of liver [79], plasma retinol declines in proportion to the hepatic concentration. These plasma retinol levels are accompanied by tissue concentrations that are low enough to result in adverse health effects ranging from xerophthalmia and nyctalopia (night blindness) to hyperkeratosis, increased susceptibility to severe infection and disturbances in cell differentiation, organ development, growth and reproduction. Xerophtalmia, along with night blindness, is one of the first manifestations of VAD and is practically pathognomonic. In pregnant women, VAD causes night blindness and increased risk of maternal mortality [80,81].

The importance of vitamin A in regulating growth through cell proliferation and differentiation was recognized early in the 20th century. Animal research results have shown that vitamin A plays a key role in mediating morphogenesis and growth of different organs. Among the first organs affected by VAD are oral cavity, tracheal mucosa and the bronchopulmonary tree, ending with eye lesions. Several years later, participation of natural retinoids in embryology and organogenesis was recognized. VAD during embryogenesis leads to fetal death or to morphological malformations, which range from organ agenesis to rudimentary or hypoplastic organs, depending on the severity of the deficiency [13,14,82,83]. This wide range of clinical manifestations can be reverted by adequate treatment with retinoids. Currently, there is sufficient experimental evidence to consider retinoids to be a hopeful promise in the prevention and therapy of several pathologies. Finally, it is worth emphasizing the importance of maintaining the concentration of retinoids within the physiological range since concentrations either above or below it cause adverse effects.

### 4.2. Epidemiology and Incidence

VAD is a major public health problem in more than half of all countries. With protein malnutrition, it is actually the most serious and commonest nutritional disorder worldwide. Recent data that reflect the incidence of VAD on the population, especially in developing countries, are most alarming [81]. In fact, it is estimated that 250 million preschool-aged children and 19 million pregnant women have biochemical VAD (serum concentration <0.7 µM), and that 5 million children are clinically affected by VAD. In developing countries, it is the leading cause of preventable blindness and is one of the most frequent causes of infant mortality. In 1987, the WHO estimated that VAD was endemic in 39 countries, based on xerophthalmia prevalence and plasma retinol levels lower than 0.35 µM. In 1995, the WHO updated these estimates and reported that VAD was a serious public health problem in over 120 countries, as over 1 million childhood deaths were associated with VAD every year. The WHO global estimates of VAD from 1995–2005 indicated that 45 and 122 countries had a public health problem of night blindness and biochemical VAD, respectively, in preschool-age children. Apart from preschool children in these countries, other groups of people at high risk for VAD are pregnant and lactating women, and higher prevalence appears in pregnant women of low social classes and in people affected by human immunodeficiency virus [80].

In the developed world, it is important to note that over 20% of the population do not reach two-thirds of recommended intake, and have lower plasma and liver concentrations of vitamin A than those accepted as normal. This situation can be aggravated by the increasingly common tendency to reduce fat intake and to engage in uncontrolled weight loss diets [84]. Other actual risk factors for VAD include stress, diseases which affect the intestine’s ability to absorb fat, infections and alcohol abuse [80,85].

In a global health policy context, a worldwide effort has been made to control VAD and related diseases in the last decade. However, vitamin A intake is still low in several countries, such as Eastern countries, where rice, which lacks this vitamin, constitutes the main part of diet. Nowadays, the various approaches to reduce VAD include promoting home gardening, health and nutrition education, fortification of commonly consumed food and supplementation for at-risk populations [80,81].

## 5. Vitamin A Deficiency and Alterations in Extracellular Matrix

Retinoid signaling participates in the expression of ECM proteins including collagen, laminin, entactin, fibronectin, elastin and proteoglycans [86,87,88,89,90]. In addition, RA also affects the expression of cell membrane ECM receptors. Consequently, vitamin A deficiency can induce changes in the composition and structure of ECM to result in alterations of organ function and pathological consequence, which could be reversed by appropriate treatment with retinoids. Excessive deposition of the ECM in tissue in response to different stimuli can occur in various organs, including kidney, lung, liver and others, leading to fibrosis, which disrupts the normal architecture of the organ to cause pathophysiological damage. This review has focused on documenting the impact of VAD on the ECM of several organs, reversibility by treatment with RA, and the importance of maintaining vitamin A tissue levels within the physiological range.

### 5.1. Kidney

Vitamin A and its biologically active derivatives have a profound influence on organ development, cell proliferation and cell differentiation, and kidney is no exception. The fact that vitamin A plays an important role in nephrogenesis was already observed in the first half of the last century by feeding pregnant rats with a diet lacking vitamin A. Their offspring were born with serious kidney abnormalities, such as renal aplasia, hypoplasia, horseshoe kidney, and ureteral abnormalities [13,91]. Later on, it was reported that even mild VAD in pregnant rats can result in a reduced number of nephrons in fetuses, leading to a permanent deficit in adulthood [92]. Recent studies, which aimed to elucidate the cellular and molecular events associated with actions of retinoids on renal development, have demonstrated their importance for growth of ureteric bud branches and in maintaining the expression of c-Ret, a receptor for neurotrophic factors which regulates essential epithelial/mesenchymal interactions during nephrogenesis [93,94,95].

RA regulates kidney organogenesis decisively by defining the number of glomeruli per kidney [96], and VAD or mutations in the RA nuclear receptors lead to abnormalities in the fetal kidney, which may predispose to hypertension in adults [91]. It has also been suggested that prenatal exposure to VAD may be a factor that contributes disproportionately to rising kidney disease incidence in disadvantaged populations [97,98]. It has also been found that VAD induces significant alterations in renal lipid metabolism and immune status, may contribute to the development of idiopathic kidney stones and may worsen renal diseases such as pyelonephritis [99,100,101]. The beneficial effect that retinoids have on several renal disorders, and their antifibrotic and cytoprotective effect on different types of renal cells [91,102], have also been demonstrated, which supports the importance of vitamin A for proper kidney development and for maintaining its integrity and function.

The BM also has a crucial effect on kidney organogenesis and cell differentiation, and mutations affecting its components lead to inherited forms of renal disease. Likewise, alterations in the BM structure or composition are associated with a variety of kidney disorders, which can lead to kidney failure [103,104]. Thereby, loss, alteration or change in the relative amount of some α-chain can cause abnormalities in the network structure of collagen IV, thus favoring the appearance of renal disease [105,106]. By way of example, post-translational defects in α3(IV), α4(IV) o α5(IV) chains, caused by mutations in their genes, are the origin of Alport syndrome [106], or the targeted deletion of the mouse *Col4a3* gene results in progressive glomerulonephritis, and leads to death by kidney failure [107]. It is also known that type IV collagen and laminin, major components of BM, suppress glomerular mesangial cells’ susceptibility to apoptosis, a process considered to mediate in the progression of glomerular inflammation to irreversible glomerulosclerosis, whereas collagen I and fibronectin, abnormally expressed in diseased glomeruli, do not [108].

As previously mentioned, retinoids affect the production of major macromolecules of the BM. Consequently, VAD can also modify the synthesis of these macromolecules by producing changes in BM composition and organization, which may underlie the pathological manifestations caused by vitamin deficiency. Since the BM plays a crucial role in kidney, it is of much interest to understand the effect of VAD on the composition of renal BM *in vivo*. Newborn male rats have been fed a vitamin A-deficient diet for 50 days [109], and exhibited an impaired renal BM structure of non-uniform increased thickness (Table 1). BM thickening is greater in tubular BM than in glomerular BM, with a 6-fold *versus* a 2-fold increase, respectively. The amount of collagen I also doubles in renal ECM and appears as irregularly distributed fibers. Strikingly, collagen I fibers have also been seen within the tubular BM. BM thickening is an early sign of pathology, which usually precedes the onset of clinical manifestations in different kidney progressive diseases, and is due to an increased deposition of BM components [110,111,112,113]. Likewise, presence of collagen I in the mesangial matrix has been strongly associated with glomerular disease [114,115]. Under the aforementioned VAD conditions, the composition of renal BM has also been modified (Table 1) to show a significant increase in the amount of both collagen IV and content of α1(IV), α4(IV), α5(IV), α6(IV) chains, but lowered in α2(IV) and α3(IV) chains. The mRNA for each α-chain varied similarly to their respective protein content, which indicates regulation at the transcriptional level of the synthesis of α(IV) collagen chains [109]. Differences in the content of collagen IV α chains have also been observed in some renal pathologies. For example, following the induction of experimental nephritis in rats, an increase in the α1(IV), α3(IV), α4(IV) chains, but no change in the amount of the α2(IV) chain, were observed in the mesangial matrix [116]. In this same model, an increased expression of the α1(IV), α2(IV), α3(IV), α4(IV) chains in the TBM has also been found during disease progression, with the higher values corresponding to α1(IV) and α4(IV) chains [105]. Increases in the α3(IV) and α4(IV) chains has been referred to in the GBM of patients with diabetic nephropathy, but not in the mesangial matrix where, in contrast, levels of the α1(IV) and α2(IV) chains rose [117]. In patients suffering from Alport's syndrome, GBMs also appeared altered as a result of the absence or reduced expression of the α3(IV), α4(IV) and α5(IV) chains, combined with variations in the expression pattern of the other α(IV) chains [118].

**Table 1:** Kidney extracellular matrix (ECM) alterations in chronic vitamin A deficiency.

ECM Component	Modification
Collagen IV (protein)	Increased
α1(IV) chain (protein and mRNA)	Increased
α2(IV) chain (protein and mRNA)	Lowered
α3(IV) chain (protein and mRNA)	Lowered
α4(IV) chain (protein and mRNA)	Increased
α5(IV) chain (protein and mRNA)	Increased
α6(IV) chain (protein and mRNA)	Increased
Matrix Metalloproteinases and Inhibitors	
MMP 2 (protein)	Lowered
MMP 9 (protein)	Lowered
TIMP 1 (protein)	Unaffected
TIMP 2 (protein)	Unaffected
Glomerular BM	Enlarged
Tubular BM	Enlarged and presence of collagen I fibrils

Data from Marín *et al.* (2005) [109]. MMP, matrix metalloproteinase; TIMP, tissue inhibitor of metalloproteinases.

In kidneys of animals exposed to a VAD diet, diminished content and proteolytic activity of metalloproteases 2 and 9 (MMP2 and MMP9) has been observed [109]. Both MMPs play an important role in the degradation and remodeling of the BM, are synthesized by different types of renal cell in response to extracellular stimuli [119,120,121] and are involved in the BM changes that occur in the development of different diseases that affect the kidney [113,122,123]. Interestingly, the amount of their tissue inhibitors 1 (TIMP1) and 2 (TIMP2) did no change under VAD conditions [109].

Although the role played by alterations in the structure and composition of BM in renal function has not been fully clarified, it is known that they are present in most kidney diseases, even before proteinuria appears. Therefore by modifying renal BMs, VAD may lead to kidney malfunction and disease.

### 5.2. Lung

As occurs in kidney, vitamin A and its biologically active derivatives have a profound influence on lung and alveolar formation during the neonatal period. They are also required for alveolar architecture maintenance and regeneration after the alveoli have been formed [124,125,126]. Pulmonary alveoli are formed early in the postnatal life and continue to form until about the age 18 months in humans or until 6–7 weeks in rats [125,127].

Lung morphogenesis is a highly regulated process which includes prenatal and postnatal stages that are dependent, at least in part, on the local vitamin A stores during active growth and differentiation. In this sense, lung reserves of vitamin A and its derivatives undergo a complicated evolution. During the last third of gestation, lungs accumulate retinyl esters, but a major depletion of such vitamin A storage molecules occurs before birth and continues after birth, and the amount of retinol and RA rises postpartum in the interstitial fibroblast [124,128].

During alveologenesis, it is known that fibroblastic, epithelial, and microvascular cells develop multiple interactions with the ECM [129]. Additionally, the ECM plays a major role in regulating alveolarization, tissue compliance, tensile and compressive strength and elasticity, tissue repair and remodeling [130]. Through specific nuclear receptors, RA modulates the expression of several ECM proteins both directly, by acting on their gene promoters, and indirectly by modifying the expression of other profibrotic factors [86,131]. Consequently, it has been described that VAD induces changes in the composition and structure of the lung ECM and the BM to result in alterations in organ function and prolonged pathological consequences. Around 50% of neonates born to vitamin A-deficient rats are born dead or die soon after birth, which may be due to pathophysiological changes in lungs [14,132]. In fact, VAD promotes bronchial hyperreactivity [133], leads to emphysematous lungs with areas of interstitial pneumonitis [134,135], and results in squamous cell metaplasia with a relatively decreased proportion of mucous and ciliated cells in the tracheobronchial tree [136]. Furthermore, the lungs of VAD rats show a reduction in the number and surface of alveoli, and alveolus septum thickening [137]. Other studies have shown that the alveolar BM in VAD rats doubles in thickness and contains irregularly scattered collagen fibrils [138]. Moreover, surfactant synthesis and ornithine decarboxylase activity, the rate-limiting enzyme in the synthesis of polyamines, are significantly lower in type II pneumocytes isolated from VAD rats [135], indicating impaired functional and proliferative capacity.

These alterations in lung function and architecture are associated with changes in ECM/BM protein content and distribution (Table 2). It has been reported that the lungs of VAD rats contain less collagen in the adventitia of small arteries and arterioles and in the alveolar septa, accompanied by a shortened length of alveolar and alveolar duct collagen fibers. However, the amount of collagen increases in the areas of interstitial pneumonitis, but remains normal in peribronchial ECM. Elastin content is also lower in not only the lung parenchyma, but also in the small arteries and arterioles of VAD rats [135,139,140]. In agreement with these findings, mild vitamin A deficiency (MVAD) in rats shows fewer elastin deposits, but effects on collagen content are even more complicated; MVAD during pregnancy leads to reduced collagen, but lung collagen deposition increases after birth with continued MVAD [137]. In our studies, in which severe and chronic VAD was induced, serum retinol contents in VAD animals were less than 0.05% of the controls, and an enlargement of the alveolar BM and the appearance of ectopic collagen fibrils in the BM were accompanied by increases in the total amount of both type I and type IV collagens (Table 2) [138]. In agreement with increased collagen IV content, its α chains were also increased if compared to the control lungs. However, this change was chain-specific. An increase in chains α1, α3 and α4 was noted without the corresponding increments in chains α2 and α5. Laminins, the other major BM component, were also evaluated. Unlike what occurs with collagen IV, laminins decreased in VAD lungs, and the protein levels of laminin α5, β1 and γ1 chains and the mRNA levels of laminin α2 and α4 chains were significantly lower. All these modifications in the chains of both collagen IV and laminin correlated with those in their corresponding mRNAs. MMP2 and MMP9, which degrade some BM macromolecules and, hence, are important contributors to the BM structure and composition, were decreased in the absence of retinoids, but two of their physiological inhibitors, TIMP1 and TIMP2, were not modified [141]. Therefore in VAD, BM degrading capacity may be diminished, which contributes to the BM alteration observed.

**Table 2:** Lung ECM alterations in chronic vitamin A deficiency.

ECM Component	Modification	Refs.
Collagen (protein)	Increased	[135]
	Lowered	[140]
Collagen I (protein)	Increased	[138]
α1(I) chain (mRNA)	Increased	[138]
α2(I) chain (mRNA)	Increased	[138]
Elastin (protein)	Lowered	[135,139]
Collagen IV (protein)	Increased	[138]
α1(IV) chain (protein and mRNA)	Increased	[141]
α2(IV) chain (protein and mRNA)	Unaffected	[141]
α3(IV) chain (protein and mRNA)	Increased	[141]
α4(IV) chain (protein and mRNA)	Increased	[141]
α5(IV) chain (protein and mRNA)	Unaffected	[141]
Laminin		
Laminin α2 chain (mRNA)	Lowered	[141]
Laminin α4 chain (mRNA)	Lowered	[141]
Laminin α5 chain (protein and mRNA)	Lowered	[141]
Laminin β1 chain (protein and mRNA)	Lowered	[141]
Laminin γ1 chain (protein and mRNA)	Lowered	[141]
Matrix metalloproteinases and inhibitors		
MMP 2 (activity)	Lowered	[141]
MMP 9 (activity)	Lowered	[141]
TIMP 1 (protein)	Unaffected	[141]
TIMP 2 (protein)	Unaffected	[141]
Alveolar BM	Enlarged and presence of collagen I fibrils	[138]

MMP, matrix metalloproteinase; TIMP, tissue inhibitor of metalloproteinases.

### 5.3. Liver

The liver is the most important tissue for vitamin A storage and metabolism, and is responsible for the regulation of retinoid homeostasis, which is accomplished by the enzymes and proteins implicated in the transport and metabolism of retinoids. In healthy individuals, approximately 90 percent of body vitamin A is stored in the liver, and is mostly concentrated in lipid droplets as retinyl ester in the hepatic stellate cells (HSC) (also known as Ito cells, lipocytes or fat-storing cells) located in the space of Disse, between the sinusoidal endothelial cells and hepatic epithelial cells. The hepatic level of vitamin A usually depends on its dietary intake and is considered the “gold standard” indicator of whole-body vitamin A status [142]. This percentage achieved in well-nourished persons may significantly lower in severely deficient individuals where kidneys and other tissues contain an appreciable amount (10%–50%) of the total-body reserve of vitamin A [143].

In addition to poor vitamin A intake, different pathological conditions, such as viral hepatitis, impaired immunity and several metabolic disorders also lower the hepatic vitamin A concentration. Xenobiotics which induce cytochrome P_450_ enzymes or other microsomal enzymes involved in retinol or RA metabolism, further contribute to hepatic retinoid depletion. Indeed, it has been reported that chronic alcohol consumption in humans causes a progressive and significant decrease in the liver retinoid concentration and that alcoholics are susceptible to develop clinical VAD symptoms [85,144]. It has also been shown in rats that a combination of ethanol and P_450_ enzyme-inducing drugs, which mimics common clinical occurrence, exacerbates hepatic vitamin A depletion [85].

The total liver volume comprises approximately 80% of parenchymal cells. These are the hepatocytes, epithelial cells which perform the majority of liver functions. Other hepatic cell types include Kupffer cells, endothelial cells, HSC (perivascular mesenchymal cells) and pit cells. The intercellular space is filled with the network of secreted locally macromolecules that constitute the ECM. The liver ECM forms part of the borderline between blood flow and hepatic parenchyma. In a normal liver, the ECM is composed of a number of macromolecules, including collagen types I, III, IV, V and VI, non-collagenous glycoproteins, which comprise laminin and fibronectin, and proteoglycans. In a normal liver sinusoid, there is a non-electron dense BM matrix which includes laminin and type IV collagen. When the liver is injured, this matrix turns into one that is rich in interstitial collagens, particularly collagens I and III [145]. Three hepatic cell types have been proved capable of the synthesis and secretion of ECM components: hepatocytes, endothelial cells and, mainly, HSCs, which are positive for a mesenchymal marker such as vimentin [146,147].

In a normal liver, HSCs represent 5%–8% of total liver cells and 15%–23% of nonparenchymal cells which, in addition to its importance as vitamin A-storing cells, are compact non-fibrogenic cells (quiescent HSC), and are especially relevant in controlling ECM turnover by not only secreting the correct amounts of ECM molecules, but by also releasing matrix MMPs and their inhibitors [148]. These cells are greatly involved in the process of hepatic disease development. Numerous *in vitro* and *in vivo* studies have shown that, when the liver is injured, HSC lose their retinoids and lipid droplets, and receive signals secreted by damaged hepatocytes and immune cells, leading them to activation and the transdifferentiation into activated myofibroblasts-like cells [147,148]. Activated myofibroblasts-like cells express alpha smooth muscle actin (α-SMA), ECM proteins and other mediators that promote liver inflammation, steatosis and a provisional fibrotic matrix deposition at the injury site to protect the liver from further damage. HSCs also express tissue TIMPs, which favors scar deposition. As α-SMA is a sensitive marker of activated HSC *in situ*, it is increasingly used as an early indicator of fibrogenic activity in human liver disease, even before the ECM accumulates [145,149,150].

Prolonged HSC activation, due to sustained hepatic injury, causes liver fibrosis characterized by both extensive scar formation and alteration of hepatic architecture, a common final stage in several chronic liver diseases which predisposes to cirrhosis and hepatocellular cancer (HCC) [145,147]. Liver cirrhosis accounts for 170,000 deaths in Europe per year, which is 1.8% of all deaths and represents the 7th commonest cause of death in western countries; eventual mortality is even higher because cirrhosis predisposes to HCC, which accounts for the vast majority of primary liver cancers [151]. The association between fibrosis and HCC is incompletely understood, but it seems likely to involve integrin signaling, interactions with the ECM, growth factors, inflammatory cells, and communication between activated HSCs and tumor cells [145].

Although the transdifferentiation of HSC to myofibroblastic cells is the central event in liver fibrogenesis, and HSCs are a major source of myofibroblasts, epithelial to mesenchymal cell transition (EMT) has also been proposed as another origin of these ECM-producing cells [145,147,151]. According to *in vitro* and *in vivo* studies, the most likely mechanism behind HSC activation, includes stimulation by TGF-β1 to promote a fibrogenic collagen-secreting phenotype and by platelet-derived growth factor, which stimulates a proliferative phenotype. TGF-β1 is not only able to drive HSCs to myofibroblastic differentiation, but may also stimulate EMT. Nevertheless, the contribution of the myofibroblasts deriving from EMT to fibrosis has been recently questioned [152]. TGF-β1 signals primarily via specific serine/threonine kinase receptors, which activate the SMAD signaling pathway. Activated SMADs, particularly SMAD3, modulates the expression of ECM proteins, including collagens I, III, V, VI and VII. Via the integration of the SMAD3 and STAT3 signaling pathways TGF-β is also a major trigger for overproduction of connective tissue growth factor (CTGF), a central mediator of ECM production, tissue remodeling and fibrosis [153].

As discussed previously, through specific nuclear receptors, RA modulates the expression of several ECM proteins, and this is an essential step in the progression of fibrogenesis and its pathobiological consequences [86,87,88]. With the liver, retinoid signaling has been reported to be involved in several pathological situations, including fibrosis, regeneration and HCC [145,154]. Progressive reduction in serum retinol and altered retinoid signaling has been noted in patients diagnosed with liver cirrhosis or HCC, and serum retinol has been proposed as a marker for HCC in high-risk groups [155]. Consequently, vitamin A deficiency may induce alterations in ECM composition and liver dysfunction. In fact, VAD is associated with the activation of HSCs in myofibroblast-like cells, enhanced cell proliferation, increased ECM deposition related with fibrogenic activation, and hepatic parenchyma disorganization. Liver parenchyma deterioration appears to be linked with loss of hepatocyte cord disposition, accumulation of fat droplets in their cytoplasm associated with increased plasma adiponectin, and elevated plasma alanine aminotransferase, a marker of liver injury [89,90]. In this sense, both functional RA loss in transgenic mice expressing the RARα-dominant negative form in hepatocytes [154] and VAD [156] induce liver damage and a lipogenic environment, which results in steatotic hepatocytes, but, contrarily, HSCs lose their lipid droplets and retinol content. This apparent paradox between hepatocytes and HSCs has been studied in alcoholic and non-alcoholic steatohepatitis models, and is explained in lipogenic regulation outcome terms. In these liver disease models, adipogenesis is up-regulated in hepatocytes, but is down-regulated in HSCs to result in lipid accumulation in hepatocytes, myofibroblastic HSC transdifferentiation and fibrogenesis [157].

The increased deposition of ECM components in livers of VAD rats has been demonstrated both *in vitro*, using isolated primary cultures of hepatocytes [158], and *in vivo*, by immunochemical methods and affects to fibronectin, laminin and type IV collagen (Table 3) [89]. An ultrastrutural analysis of VAD livers has shown the appearance of α-SMA-positive HSCs, which evidences that HSC activation takes place [89]. Accordingly, low hepatic vitamin A content, which can result not only from low dietary intake, but also from interference with vitamin A metabolism by agents such as ethanol, is a risk factor for the development of liver fibrosis, and both hepatocytes and stellate cells are involved [85,159].

**Table 3:** Liver ECM alterations in chronic vitamin A deficiency.

ECM Component	Modification
Collagen (protein)	Increased
Collagen IV (protein)	Increased
Laminin 1 (protein)	Increased
Fibronectin (protein)	Increased
Perisinusoidal space	Enhanced extracellular material

Data from Aguilar *et al.* (2009) [89].

## 6. Mechanism of Extracellular Matrix Modifications in Vitamin A Deficiency

It is becoming clear that the actions of retinoids are much more complex than previously thought. As we mention above, through several families of nuclear receptors (RARs, RXRs and PPARs), RA regulates the transcription of a battery of target genes, such as those for several growth and transcription factors. RA also has extra-nuclear, non-genomic effects, including the activation or inhibition of protein kinases from other signaling pathways. Retinol is also able to activate the Janus kinase/STAT5 signaling pathway. Therefore, VAD may affect ECM by different mechanisms. Alterations of the ECM as a result of VAD appear in every tissue analyzed, and similarities in its effects are observed, such as BM thickening, increase in collagens I and IV, and a decrease in MMPs, which suggest a common mechanism for ECM alterations in all VAD tissues. However, the modifications induced by VAD in either collagen IV chain composition, mentioned previously, or in cytokine content, TNFα increases in VAD kidney but not in lung, vary between tissues. These differences may indicate that tissue-specific mechanisms also take place in ECM modifications.

The mechanisms through which VAD alters ECM are not satisfactorily known and several possibilities, which can act concertedly or independently, may be considered. First, VAD lowers the tissue concentration of retinoids and RA can regulate the transcription of ECM proteins and their degrading proteinases directly through RARs and RXRs. It has been shown that RA inhibits the expression of both collagen I chains, α1(I) and α2(I), through the binding of the RAR-RXR heterodimer to RAREs in their gene promoters [160,161]. On the contrary, RA has a direct activating effect on the transcription of collagen X, a protein unique to the hypertrophic cartilage matrix [162]. The transcription of the β1 subunit of laminin, a major basement membrane protein, is also activated by the RARs bound to the gene promoter in response to RA [86]. The expression of the collagenases that can degrade interstitial collagen and modify ECM, such as MMP1, is inhibited by the interaction of RA-activated RARs with the collagenase promoter [163]. MMP9 up-regulation through the binding of RARα together with transcription coactivator P300 to the MMP9 gene promoter, and despite the absence of a consensus RARE, has also been described in both rat mammary glands after weaning and murine dendritic myeloid cells after RA treatment [164,165]. Second, RA regulates the expression of the cytokines involved in ECM protein synthesis and degradation, such as TGF-β1, a key molecule in the activation of the fibrotic program [166,167,168]. Via the SMAD signaling pathway, TGF-β1 can up-regulate the expression of several collagens, such as collagen I, III, V, VI and VII. Some other ECM proteins, like fibronectin and perlecan, are induced by TGF-β1 via non-SMAD pathways and others, including nidogen, laminin, versican, decorin, tenascin and several MMPs, by still non-characterized pathways [169]. TGF-β also stimulates HSC to myofibroblast transdifferentiation, induces EMT [170,171,172] and triggers the synthesis of CTGF [153], a potent inducer of ECM synthesis. All these processes potentially lead to ECM overproduction. Increased TGF-β1 levels have been found in VAD tissues, such as kidney [101], lung [138], and aorta [173]. Therefore, signaling by this profibrotic factor can also mediate in ECM modifications. Third, it is known that RA receptors can directly interact with and modify the transcriptional activity of other transcription factors, such as Sp1, thus regulating the expression of the genes lacking canonical RARES. It has been reported that RA-activated RAR-RXR heterodimers induce the expression of TGF-β1 and its receptors by physically interacting with Sp1. This interaction strengthens Sp1’s affinity to the functional GC box motifs in the promoters and enhances the transactivation of the corresponding genes [174]. A similar mechanism might operate in the expression of the ECM proteins whose promoters do not interact directly with RA receptors, but are able to bind Sp1 specifically; e.g., common promoter col4α1-col4α2 [175]. RA also cross-talks with other signaling pathways which affect the expression of ECM-related proteins. By way of example, both RA treatment and cell attachment to collagen IV inhibit the activity of ERK1, one of the terminal effectors of the MAPK pathway, to result in a lowered MMP9 expression in human carcinoma cell lines [176]. Fourth, it is known that VAD is a condition which potentially leads to an imbalance between ROS production and antioxidant defenses [138,177]. Clinical and experimental data suggest that oxidative stress-related molecules may act as mediators of the molecular and cellular events implicated in fibrosis. Reactive oxygen species (ROS), acting as secondary intracellular messengers, have been seen to activate transcription factors like AP-1, which is involved in collagen expression, and to induce TGF-β1. In fact, treating human mesangial cells with hydrogen peroxide increases the expression of ECM proteins, such as collagen types I, III and IV, and also fibronectin, mediated by TGF-β1, whose synthesis is also induced [178]. In turn, TGF-β1 stimulates ROS production in various cell types, including endothelial cells, epithelial cells, smooth muscle cells and fibroblasts [179]. ROS are also mediators of the TGF-β1-induced up-regulation of plasminogen activator inhibitor-1, a serpin considered a major inhibitor of ECM degradation and an important player in renal fibrosis [180,181]. Fifth, the interaction of the retinol-RBP complex with cell surface receptor STRA6 activates the JAK2/STAT5 signaling pathway [77]. The involvement of the JAK/STAT1/3 pathway in collagen expression and fibrotic processes has been reported [182,183,184,185], but the effects of retinol-RBP/JAK2/STAT5 pathway activation on the structure and composition of ECM have not yet been characterized. Finally, through their plasma membrane receptors, ECM molecules activate and/or interact with intracellular signaling pathways which may affect ECM. Therefore, the VAD-induced modifications in ECM molecular composition may, in turn, modulate ECM metabolism. By way of example, in human breast cancer cells, collagen IV induces the phosphorylation, nuclear translocation and DNA binding of STAT5 as well as the secretion of MMP9 [186,187]. The discoidin domain receptor 1, a receptor for collagens type I to V, also inhibits STAT1/3 activation triggered by α2β1-integrin, another ECM-activated receptor, by up-regulating the tyrosine phosphatase activity of SHP2 [57]. Moreover, collagen I induces EMT in lung cancer cells through a TGF-β-dependent mechanism, probably involving phosphoinositide-3-kinase and ERK [188].

The complexity and multiplicity of retinoid actions, together with the effects of a changing ECM, make it difficult to elucidate the mechanisms of ECM modification by VAD, and more studies are required to understand them satisfactorily.

## 7. Conclusions

The ECM is an intricate network of macromolecules which occupy the extracellular space in any tissue and organ in close association with the surface of the cell that produced them. In addition to stabilizing the physical structure of tissues, the ECM can provide specific signals through an interaction with the cell surface. ECM modifications will potentially compromise organ function and eventually lead to disease. The structure and composition of the ECM are modified in VAD and the altered ECM can act as a contributory factor in the deleterious effects of vitamin deficiency. Extracellular matrix accumulation is the most prominent characteristic in the pathogenesis of fibrotic disease. Liver, pulmonary and renal fibrosis are common pathological states which represent a major global health problem, and effective therapeutic measures are presently unavailable [189]. The similarities in the ECM modifications occurring in VAD and in some other diseases, and the reversibility of most VAD-induced alterations by RA, suggest that some degree of VAD might be present in those diseases and underscores the potentially beneficial effects of retinoid treatment [109,138,141,168,190,191,192,193,194,195]. In fact, RA treatment has proved beneficial in several experimental diseases. In an experimental anti-glomerular BM glomerulonephritis model, administration of all-*trans*-RA reduces proteinuria and the number of affected glomeruli [196]. RA administration to VAD animals also restores the concentration of parenchymal elastic fibers and lung mechanical properties [139,140]. Moreover, RA administration to experimental animals causes a marked reduction in type I collagen mRNA in both total hepatic and purified Ito cell RNA, inhibits the expression of TGF-β1 and CTGF, and can play a protective role against cell injury, proliferation and collagen accumulation [197,198]. Although there are some reports that show adverse effects of RA in fibrogenesis, nowadays there is sufficient experimental evidence to consider retinoids a hopeful promise in the prevention and therapy of several pathologies, including those affecting the ECM [88,189].

## References

[1] McLaren D.S., Kraemer K. (2012). Vitamin A in nature. World Rev. Nutr. Diet.

[2] De Luca L.M. (1991). Retinoids and their receptors in differentiation, embryogenesis and neoplasia. FASEB J..

[3] Duester G. (2008). Retinoic acid synthesis and signaling during early organogenesis. Cell.

[4] Mark M., Ghyselinck N.B., Chambon P. (2009). Function of retinoic acid receptors during embryonic development. Nucl. Recept. Signal..

[5] Ross A.C. (2012). Vitamin A and retinoic acid in T cell-related immunity. Am. J. Clin. Nutr..

[6] Sommer A., Vyas K.S. (2012). A global clinical view on vitamin A and carotenoids. Am. J. Clin. Nutr..

[7] Rhinn M., Dolle P. (2012). Retinoic acid signaling during development. Development.

[8] Gudas L.J. (2013). Retinoids induce stem cell differentiation via epigenetic changes. Semin. Cell Dev. Biol..

[9] Chambon P. (1996). A decade of molecular biology of retinoic acid receptors. FASEB J..

[10] Perissi V., Jepsen K., Glass C.K., Rosenfeld M.G. (2010). Deconstructing repression: Evolving models of corepressor action. Nat. Rev. Genet..

[11] Ziouzenkova O., Orasanu G., Sharlach M., Akiyama T.E., Berger J.P., Viereck J., Hamilton J.A., Tang G., Dolnokowski G.G., Vogel S. (2007). Retnaldehyde represses adipogenesis and diet-induced obesity. Nat. Med..

[12] Berry D.C., Noy N. (2012). Signaling by vitamin A and retinol-binding protein in regulation of insulin responses and lipid homeostasis. Biochim. Biophys. Acta.

[13] Wilson J.G., Roth C.B., Warkany J. (1953). An analysis of the syndrome of malformations induced by maternal vitamin A deficiency. Effects of restoration of vitamin A at various times during gestation. Am. J. Anat..

[14] Takahashi Y.I., Smith J.E., Winick M., Goodman D.S. (1975). Vitamin A deficiency and fetal growth and development in the rat. J. Nutr..

[15] Frantz C., Stewart K.M., Weaver V.M. (2010). The extracellular matrix at a glance. J. Cell Sci..

[16] Hynes R.O., Naba A. (2012). Overview of the matrisome-an inventory of extracellular matrix constituents and functions. Cold Spring Harb. Perspect. Biol..

[17] Alam N., Goel H.L., Zarif M.J., Butterfield J.E., Perkins H.M., Sansoucy B.G., Sawyer T.K., Languino L.R. (2007). The integrin-growth factor receptor duet. J. Cell. Physiol..

[18] Kim S.H., Turnbull J., Guimond S. (2011). Extracellular matrix and cell signaling: The dynamic cooperation of integrin, proteoglycan and growth factor receptor. J. Endocrinol..

[19] Van Dijk M., Göransson S.A., Strömblad S. (2013). Cell to extracellular matrix interactions and their reciprocal nature in cancer. Exp. Cell Res..

[20] Järveläinen H., Sainio A., Koulu M., Wight T.N., Penttinen R. (2009). Extracellular matrix molecules: Potential targets in pharmacotherapy. Pharmacol. Rev..

[21] Timpl R. (1989). Structure and biological activity of basement membrane proteins. Eur. J. Biochem..

[22] Ricard-Blum S. (2011). The collagen family. Cold Spring Harb. Perspect. Biol..

[23] Baldwin A.K., Simpson A., Steer R., Cain S.A., Kielty C.M. (2013). Elastic fibers in health and disease. Exp. Rev. Mol. Med..

[24] Tsang K.Y., Cheung M.C.H., Chan D., Cheah K.S.E. (2010). The developmental roles of the extracellular matrix: Beyond structure to regulation. Cell Tissue Res..

[25] Pankov R., Yamada K.M. (2002). Fibronectin at a glance. J. Cell Sci..

[26] Ffrench-Constant C. (1995). Alternative splicing of fibronectin-many different proteins but few different functions. Exp. Cell Res..

[27] Mao Y., Schwarzbauer J.E. (2005). Fibronectin fibrillogenesis, a cell-mediated matrix assembly process. Matrix Biol..

[28] Singh P., Carraher C., Schwarzbauer J.E. (2010). Assembly of fibronectin extracellular matrix. Annu. Rev. Cell Dev. Biol..

[29] LeBleu V.S., MacDonald B., Kalluri R. (2007). Structure and function of basement membranes. Exp. Biol. Med..

[30] Hudson B.G., Tryggvason K., Sundaramoorthy M., Neilson E.G. (2003). Alport’s syndrome, Goodpasture’s syndrome, and type IV collagen. N. Engl. J. Med..

[31] Zhou J., Ding M., Zhao Z., Reeders S.T. (1994). Complete primary structure of the sixth chain of human basement membrane collagen, α6(IV). Isolation of the cDNAs for α6(IV) and comparison with five other type IV collagen chains. J. Biol. Chem..

[32] Khoshnoodi J., Pedchenko V., Hudson B.G. (2008). Mammalian collagen IV. Microsc. Res. Tech..

[33] Timoneda J., Gunwar S., Monfort G., Saus J., Noelken M., Hudson B.G. (1990). Unusual dissociative behaviour of the noncollagenous domain (hexamer) of basement membrane collagen during electrophoresis and chromatofocusing. Connect. Tissue Res..

[34] Sundaramoorthy M., Meiyappan M., Todd P., Hudson B.G. (2002). Crystal structure of NC1 domains: Structural basis for type IV collagen assembly in basement membranes. J. Biol. Chem..

[35] Khoshnoodi J., Cartailler J.P., Alvares K., Veis A., Hudson B.G. (2006). Molecular recognition in the assembly of collagens: Terminal non-collagenous domains are key recognition modules in the formation of triple-helical protomers. J. Biol. Chem..

[36] Khoshnoodi J., Sigmundsson K., Cartailler J.P., Bondar O., Sundaramoorthy M., Hudson B.G. (2006). Mechanism of chain selection in the assembly of collagen IV: A prominent role for the α2 chain. J. Biol. Chem..

[37] Yurchenco P.D. (2011). Basement membranes: Cell scaffoldings and signaling platforms. Cold Spring Harb. Perspect. Biol..

[38] Hohenester E., Yurchenco P.D. (2013). Laminins in basement membrane assembly. Cell Adh. Migr..

[39] Cheng Y.S., Champliaud M.F., Burgeson R.E., Marinkovich M.P., Yurchenco P.D. (1997). Self-assembly of laminin isoforms. J. Biol. Chem..

[40] Iozzo R.V., Zoeller J.J., Nyström A. (2009). Basement membrane proteoglycans: Modulators par excellence of cancer growth and angiogenesis. Mol. Cells.

[41] Whitelock J.M., Melrose J., Iozzo R.V. (2008). Diverse cell signaling events modulated by perlecan. Biochemistry.

[42] Gill S., Wight T.N., Frevert C.W. (2010). Proteoglycans: Key regulators of pulmonary inflammation and the innate immune response to lung infection. Anat. Rec..

[43] Ho M.S.P., Böse K., Mokkapati S., Nischt R., Smyth N. (2008). Nidogens-Extracellular matrix linker molecules. Microsc. Res. Tech..

[44] McKee K.K., Harrison D., Capizzi S., Yurchenco P.D. (2007). Role of laminin terminal globular domains in basement assembly. J. Biol. Chem..

[45] Behrens D.T., Villone D., Koch M., Brunner G., Sorokin L., Robenek H., Bruckner-Tuderman L., Bruckner P., Hansen U. (2012). The epidermal basement membrane is a composite of separate laminin- or collagen IV-containing networks connected by aggregated perlecan, but not by nidogens. J. Biol. Chem..

[46] Campbell I.D., Humphries M.J. (2011). Integrin structure, activation, and interactions. Cold Spring Harb. Perspect. Biol..

[47] Humpries J.D., Byron A., Humphries J. (2006). Integrin ligands. J. Cell Sci..

[48] Giancotti F.G., Ruoslahti E. (1999). Integrin signaling. Science.

[49] Lock J.G., Wehrle-Haller B., Strömblad S. (2008). Cell-matrix adhesion complexes: Master control machinery of cell migration. Semin. Cancer Biol..

[50] Legate K.R., Wickström S.A., Fässler R. (2009). Genetic and cell biological analysis of integrin outside-in signaling. Genes Dev..

[51] Spence H.J., Dhillon A.S., James M., Winder S.J. (2004). Dystroglycan, a scaffold for the ERK-MAP kinase cascade. EMBO Rep..

[52] Moore C.J., Winder S.J. (2012). The inside and out of dystroglycan post-translational modification. Neuromuscul. Disord..

[53] Vogel W.F., Abdulhussein R., Ford C.E. (2006). Sensing extracellular matrix: An update on discoidin domain receptor function. Cell Signal..

[54] Leitinger B. (2011). Transmembrane collagen receptors. Annu. Rev. Cell Dev. Biol..

[55] Vogel W., Gish G.D., Alves F., Pawson T. (1997). The discoidin domain receptor tyrosine kinases are activated by collagen. Mol. Cell.

[56] Shrivastava A., Radziejewski C., Campbell E., Kovac L., McGlynn M., Ryan T.E., Davis S., Goldfarb M.P., Glass D.J., Lemke G. (1997). An orphan receptor tyrosine kinase family whose members serve as nonintegrin collagen receptors. Mol. Cell.

[57] Wang C.Z., Su H.W., Hsu Y.C., Shen M.R., Tang M.J. (2006). A discoidin domain receptor 1/SHP-2 signaling complex inhibits α2β1-integrim-mediated signal transducers and activators of transcription 1/3 activation and cell migration. Mol. Biol. Cell.

[58] Xu H., Bihan D., Chang F., Huang P.H., Farndale R.W., Leitinger B. (2012). Discoidin domain receptors promote α1β1- and α2β1-integrin mediated cell adhesion to collagen by enhancing integrin activation. PLoS One.

[59] Dejmek J., Dib K., Jönsson M., Andersson T. (2003). Wnt-5a and G-protein signaling are required for collagen-induced DDR1 receptor activation and normal mammary cell adhesion. Int. J. Cancer.

[60] Kim H.G., Hwang S.Y., Aaronson S.A., Mandinova A., Lee S.W. (2011). DDR1 receptor tyrosine kinase promotes prosurvival pathway through Notch1 activation. J. Biol. Chem..

[61] Iwai L.K., Chang F., Huang P.H. (2013). Phosphoproteomic analysis identifies insulin enhancement of discoidin domain receptor 2 phosphorylation. Cell Adh. Migr..

[62] Fu H.L., Valiathan R.R., Arkwright R., Sohail A., Mihai C., Kumarasi M., Mahasenan K.V., Mobashery S., Huang P., Agarwal G. (2013). Discoidin domain receptors: Unique receptor tyrosine kinases in collagen-mediated signaling. J. Biol. Chem..

[63] Jandrot-Perrus M., Busfield S., Lagrue A.H., Xiong X., Debili N., Chickering T., Le Couedic J.P., Goodearl A., Dussault B., Fraser C. (2000). Cloning, characterization, and functional studies of human and mouse glycoprotein VI: A platelet-specific collagen receptor from the immunoglobulin superfamily. Blood.

[64] Lebbink R.J., de Ruiter T., Adelmeijer J., Brenkman A.B., van Helvoort J.M., Koch M., Farndale R.W., Lisman T., Sonnenberg A., Lenting P.J. (2006). Collagens are functional, high affinity ligands for the inhibitory immune receptor LAIR-1. J. Exp. Med..

[65] Meyaard L. (2010). LAIR and collagens in immune regulation. Immunol. Lett..

[66] Watson S.P., Herbert J.M.J., Pollitt A.Y. (2010). GPVI and CLEC-2 in hemostasis and vascular integrity. J. Thromb. Haemost..

[67] Berry D.C., Noy N. (2009). All-trans-retinoic acid represses obesity and insulin resistance by activating both peroxisome proliferation-activated receptor β/δ and retinoic acid receptor. Mol. Cell. Biol..

[68] Chen J.Y., Clifford J., Zusi C., Starrett J., Tortolani D., Ostrowski J., Reczek P.R., Chambon P., Gronemeyer H. (1996). Two distinct actions of retinoid-receptor ligands. Nature.

[69] Bruck N., Vitoux D., Ferry C., Duong V., Bauer A., de The H., Rochette-Egly C. (2009). A coordinated phosphorylation cascade initiated by p38MAPK/MSK1 directs RARα to target promoters. EMBO J..

[70] Al Tanoury Z., Piskunov A., Rochette-Egly C. (2013). Vitamin A and retinoid signaling: Genomic and non-genomic effects. J. Lipid Res..

[71] Cañón E., Cosgaya J.M., Scsucova S., Aranda A. (2004). Rapid effects of retinoic acid on CREB and ERK phosphorylation in neuronal cells. Mol. Biol. Cell.

[72] Masiá S., Alvarez S., de Lera A.R., Barettino D. (2007). Rapid, non-genomic actions of retinoic acid on phosphatidylinositol-3-kinase signaling pathway mediated by the retinoic acid receptor. Mol. Endocrinol..

[73] Zhao Q., Tao J., Zhu Q., Jia P.-M., Dou A.-X., Li X., Cheng F., Waxman S., Chen G.-Q., Chen S.-J. (2004). Rapid induction of cAMP/PKA pathway during retinoic acid-induced acute promyelocytic leukemia cell differentiation. Leukemia.

[74] Kambhampati S., Li Y., Verma A., Sassano A., Majchrzak B., Deb D.K., Parmar S., Giafis N., Kalvakolanu D.V., Rahman A. (2003). Activation of protein kinase Cδ by all-trans-retinoic acid. J. Biol. Chem..

[75] Pendaries V., Verrecchia F., Michel S., Mauviel A. (2003). Retinoic acid receptors interfere with the TGF-β/Smad signaling Pathway in a ligand-specific manner. Oncogene.

[76] Cao Z., Flanders K.C., Bertolette D., Lyakh L.A., Wurthner J.U., Parks T.W., Letterio J.J., Ruscetti F.W., Roberts A.B. (2003). Levels of phosphor-Smad2/3 are sensors of the interplay between effects of TGF-β and retinoic acid on monocytic and granulocytic differentiation of HL-60 cells. Blood.

[77] Berry D.C., Jin H., Majumdar A., Noy N. (2011). Signaling by vitamin A and retinol-binding protein regulates gene expression to inhibit insulin responses. Proc. Natl. Acad. Sci. USA.

[78] Petrosino J.M., Disilvestro D., Ziouzenkova O. (2014). Aldehyde dehydrogenase 1A1: Friend or foe to female metabolism?. Nutrients.

[79] National Academy of Sciences (NAS) (2006). The Essential Guide to Nutrient Requirements. Food and Nutrition Board.

[80] World Health Organization (WHO) (2009). Global prevalence of vitamin A deficiency in populations at risk 1995–2005. WHO Global Database on Vitamin A Deficiency.

[81] World Health Organization (WHO) (2014). Micronutrients Deficiencies.

[82] Wolbach S.B., Howe P.R. (1925). Tissue changes following deprivation of fat soluble A vitamin. J. Exp. Med..

[83] Underwood B.A., Sporn M.B., Roberts A., Goodman D.S. (1984). The Retinoids.

[84] Bendich A., Langseth L. (1989). Safety of vitamin A. Am. J. Clin. Nutr..

[85] Lieber C.S. (2000). Alcohol: Its metabolism and interaction with nutrients. Annu. Rev. Nutr..

[86] Matsui T. (1996). Differential activation of the murine laminin B1 gene promoter by RARα, RORα, and AP-1. Biochem. Biophys. Res. Commun..

[87] Axel D.I., Frigge A., Dittmann J., Runge H., Spyridopoulps I., Riessen R., Viebahn R., Karsch K.R. (2001). All-trans retinoic acid regulates proliferation, migration, differentiation, and extracellular matrix turnover of human arterial smooth muscle cells. Cardiovasc. Res..

[88] Lee Y.S., Jeong W.I. (2012). Retinoic acids and hepatic stellate cells in liver disease. J. Gastroenterol. Hepatol..

[89] Aguilar R.P., Genta S., Oliveros L., Anzulovich A., Giménez M.S., Sánchez S.S. (2009). Vitamin A deficiency injures liver parenchyma and alters the expression of hepatic extracellular matrix. J. Appl. Toxicol..

[90] Barber T., Esteban-Pretel G., Marín M.P., Timoneda J., Preedy V.R. (2012). Vitamin A and Carotenoids: Chemistry, Analysis, Function and Effects.

[91] Xu Q., Lucio-Cazana J., Kitamura M., Ruan X., Fine L.G., Norman J.T. (2004). Retinoids in nephrology: Promises and pitfalls. Kidney Int..

[92] Lelièvre-Pégorier M., Vilar J., Ferrier M.L., Moreau E., Freund N., Gilbert T., Merlet-Bénichou C. (1998). Mild vitamin A deficiency leads to inborn nephron deficit in the rat. Kidney Int..

[93] Moreau E., Vilar J., Lelièvre-Pégorier M., Merlet-Benichou C., Gilbert T. (1998). Regulation of c-ret expression by retinoic acid in rat metanephros: Implication in nephron mass control. Am. J. Physiol..

[94] Mendelsohn C., Batourina E., Fung S., Gilbert T., Dodd J. (1999). Stromal cells mediated retinoid-dependent functions essential for renal development. Development.

[95] Batourina E., Gim S., Bello N., Shy M., Clagett-Dame M., Srinivas S., Costantini F., Mendelsohn C. (2001). Vitamin A controls epithelial/mesenchymal interactions through Ret expression. Nat. Genet..

[96] Vilar J., Gilbert T., Moreau E., Merlet-Bénichou C. (1996). Metanephros organogenesis is highly stimulated by vitamin A derivatives in organ culture. Kidney Int..

[97] Merlet-Bénichou C., Vilar J., Lelièvre-Pégorier M., Gilbert T. (1999). Role of retinoids in renal development: Pathophysiological implication. Curr. Opin. Nephrol. Hypertens..

[98] Nelson R.G. (2003). Intrauterine determinants of diabetic kidney disease in disadvantaged populations. Kidney Int..

[99] Atadzhanov U.Zh., Utegeov N.U. (2003). Structural-functional damage to cellular membranes in deficiency of vitamins A, E, B2, PP in children with calculous pyelonephritis. Urologiia.

[100] Sakly R., Fekih M., Ben Amor A., Najjar M.F., Mbazaa M. (2003). Possible role of vitamin A and E deficiency in human idiopathic lithiasis. Ann. Urol..

[101] Yang H., Chen K., Zhang X., Wang L., Li C., Tao H., Wang L., Li Q. (2010). Vitamin A deficiency results in dysregulation of lipid efflux pathway in rat kidney. Pediatr. Nephrol..

[102] Wagner J. (2001). Potential role of retinoids in the therapy of renal disease. Nephrol. Dial. Transplant..

[103] Müller U., Brändli A.W. (1999). Cell adhesion molecules and extracellular matrix constituents in kidney development and disease. J. Cell Sci..

[104] Gustafsson E., Fassler R. (2000). Insights into extracellular matrix functions from mutant mouse models. Exp. Cell. Res..

[105] Van Vliet A., Van Alderwegen I.E., Baelde H.J., de Heer E., Killen P.D., Kalluri R.K., Bruijn J.A., Bergijk E.C. (1999). Differential expression of collagen type IV alpha-chains in the tubulointerstitial compartment in experimental chronic serum sickness nephritis. J. Pathol..

[106] Hudson B.G. (2004). The molecular basis of goodpasture and alport syndromes: Beacons for the Discovery of the collagen IV family. J. Am. Soc. Nephrol..

[107] Miner J.H., Sanes J.R. (1996). Molecular and functional defects in kidneys of mice lacking collagen 3 (IV): Implications for Alport syndrome. J. Cell Biol..

[108] Mooney A., Jackson K., Bacon R., Strenli C., Edwards G., Bassuk J., Savill J. (1999). Type IV collagen and laminin regulate glomerular mesangial cell susceptibility to apoptosis via 1 integrin-mediated survival signals. Am. J. Pathol..

[109] Marín M.P., Esteban-Pretel G., Alonso R., Sado Y., Barber T., Renau-Piqueras J., Timoneda J. (2005). Vitamin A deficiency alters the structure and collagen IV composition of rat renal basement membranes. J. Nutr..

[110] Yang C.W., Hattori M., Vlassara H., He C.J., Carome M.A., Yamato E., Elliot S., Striker G.E., Striker L.J. (1995). Overexpression of transforming growth factor-beta 1 mRNA is associated with upregulation of glomerular tenascin and laminin gene expression in nonobese dibetic mice. J. Am. Soc. Nephrol..

[111] Sayers R., Kalluri R., Rodgers K.D., Shield C.F., Meehan D.T., Cosgrove D. (1999). Role of transforming growth factor-beta1 in Alport renal disease progression. Kidney Int..

[112] Noel L.H. (2000). Renal pathology and ultrastructural findings in Alport’s syndrome. Ren. Fail..

[113] Tsilibary E.C. (2003). Microvascular basement membranes in diabetes mellitus. J. Pathol..

[114] Striker L.J., Killen P.D., Chi E., Striker G.E. (1984). The composition of glomerulosclerosis I. Studies in focal sclerosis, cresenteric glomerulonephritis and membranoproliferative glomerulonephritis. Lab. Investig..

[115] Ebihara I., Suzuk S., Nakamura T., Fukui M., Yaguchi Y., Tomino Y., Koide H. (1993). Extracellular matrix component mRNA expression in glomeruli in experimental focal glomerulosclerosis. J. Am. Soc. Nephrol..

[116] Bergijk E.C., van Alderwegen I.E., Baelde H.J., de Heer E., Funabiki K., Miyai H., Killen P.D., Kalluri R.K., Bruijn J.A. (1998). Differential expression of collagen IV isoforms in experimental glomerulosclerosis. J. Pathol..

[117] Yagame M., Kim Y., Zhu D., Suzuki D., Eguchi K., Nomoto Y., Sakai H., Groppoli T., Steffes M.W., Mauer S.M. (1995). Differential distribution of type IV collagen chains in patients with diabetic nephropathy in non-insulin dependent diabetes mellitus. Nephron.

[118] Barsotti P., Muda A.O., Mazzucco G., Massella L., Basolo B., de Marchi M., Rizzoni G., Monga G., Faraggiana T. (2001). Distribution of a-chains of type I V collagen in glomerular basement membranes with ultrastructural alterations suggestive of Alport syndrome. Nephrol. Dial. Transplant..

[119] McLennan S.V., Martel S.K., Yue D.K. (2002). Effects of mesangium glycation on matrix metalloproteinase activities: Possible role in diabetic nephropathy. Diabetes.

[120] Akool El-S., Kleinert H., Hamada F.M., Abdelwahab M.H., Forstermann U., Pfeilschifter J., Eberhardt W. (2003). Nitric oxide increases the decay of matrix metalloproteinase 9 mRNA by inhibiting the expression of mRNA-stabilizing factor HuR. Mol. Cell. Biol..

[121] Cheng S., Lovett D.H. (2003). Gelatinase A (MMP-2) is necessary and sufficient for renal tubular cell epithelial-mesenchymal transformation. Am. J. Pathol..

[122] Camp T.M., Smiley L.M., Hayden M.R., Tyagi S.C. (2003). Mechanism of matrix accumulation and glomerulosclerosis in spontaneously hypertensive rats. J. Hypertens..

[123] Rao V.H., Lee G.E., Kashtan C.E., Nemori R., Singh R.K., Meehan D.T., Rodgers K., Berridge B.R., Bhattacharya G., Cosgrove D. (2003). Increased expression of MMP-2, MMP-9 (type IV collagenases/gelatinases), and MT1-MMP in canine X-linked Alport syndrome (XLAS). Kidney Int..

[124] Chytil F. (1996). Retinoids in lung development. FASEB J..

[125] Massaro D., Massaro G.D. (2002). Pulmonary alveoli: Formation, “the call for oxygen”, and other regulators. Am. J. Physiol. Lung Cell. Mol. Physiol..

[126] Maden M., Hind M. (2004). Retinoic acid in alveolar development, maintenance and regeneration. Philos. Trans. R. Soc. Lond. B Biol. Sci..

[127] Zeltner T.B., Caduff J.H., Gehr P., Pfenninger J., Burri P.H. (1987). The postnatal development and growth of the human lung. I. Morphometry. Respir. Physiol..

[128] Shenai J.P., Chytil F., Stahlman M.T. (1985). Vitamin A status of neonates with bronchopulmonary dysplasia. Pediatr. Res..

[129] Herzog E.L., Brody A.R., Colby T.V., Mason R., Williams M.C. (2008). Knowns and unknowns of the alveolus. Proc. Am. Thorac. Soc..

[130] Pelosi P., Rocco P.R., Negrini D., Passi A. (2007). The extra- cellular matrix of the lung and its role in edema formation. An. Acad. Bras. Cienc..

[131] Salbert G., Fanjul A., Piedrafita F.J., Lu X.P., Kim S.J., Tran P., Pfahl M. (1993). Retinoic acid receptors and retinoid X receptor-α down-regulate the transforming growth factor-β1 promoter by antagonizing AP-1 activity. Mol. Endocrinol..

[132] Antipatis C., Ashworth C.J., Grant G., Lea R.G., Hay S.M., Rees W.D. (1998). Effects of maternal vitamin A status on fetal heart and lung: Changes in expression of key developmental genes. Am. J. Physiol..

[133] McGowan S.E., Smith J., Holmes A.J., Smith L.A., Businga T.R., Madsen M.T., Kopp U.C., Kline J.N. (2002). Vitamin A deficiency promotes bronchial hyperreactivity in rats by altering muscarinic M2 receptor function. Am. J. Physiol. Lung Cell. Mol. Physiol..

[134] Baybutt R.C., Molteni A. (2007). Vitamin A and emphysema. Vitam. Horm..

[135] Baybutt R.C., Hu L., Molteni A. (2000). Vitamin A deficiency injures lung and liver parenchyma and impairs function of rat type II pneumocytes. J. Nutr..

[136] Biesalski H.K., Nohr D. (2003). Importance of vitamin-A for lung function and development. Mol. Aspects Med..

[137] Wei H., Huang H.M., Li T.Y., Qu P., Liu Y.X., Chen J. (2009). Marginal vitamin A deficiency affects lung maturation in rats from prenatal to adult stage. J. Nutr. Sci. Vitaminol. Tokyo.

[138] Esteban-Pretel G., Marín M.P., Renau-Piqueras J., Barber T., Timoneda J. (2010). Vitamin A deficiency alters rat lung alveolar basement membrane: Reversibility by retinoic acid. J. Nutr. Biochem..

[139] McGowan S.E., Takle E.J., Holmes A.J. (2005). Vitamin A deficiency alters the pulmonary parenchymal elastic modulus and elastic fiber concentration in rats. Respir. Res..

[140] McGowan S.E., Holmes A.J. (2007). Vitamin A deficiency alters pulmonary parenchymal collagen and tissue mechanics. Respir. Physiol. Neurobiol..

[141] Esteban-Pretel G., Marín M.P., Renau-Piqueras J., Sado Y., Barber T., Timoneda J. (2013). Vitamin A deficiency disturbs collagen IV and laminin composition and decreases matrix metalloproteinase concentrations in rat lung. Partial reversibility by retinoic acid. J. Nutr. Biochem..

[142] Ross C., Zolfaghari R. (2004). Regulation of hepatic retinol metabolism: Perspectives from studies on vitamin A status. J. Nutr..

[143] Olson J.A. (1987). Recommended dietary intakes (RDI) of vitamin A in Humans. Am. J. Clin. Nutr..

[144] Clugston R.D., Blaner W.S. (2012). The adverse effects of alcohol on vitamin A metabolism. Nutrients.

[145] Iredale J.P., Thompson A., Henderson N.C. (2013). Extracellular matrix degradation in liver fibrosis: Biochemistry and regulation. Biochim. Biophys. Acta.

[146] Clement B., Grimaud J.A., Campion J.P., Deugnier Y., Guillouzo A. (1986). Cell types involved in collagen and fibronectin production in normal and fibrotic human liver. Hepatology.

[147] Friedman S.L. (2000). Molecular regulation of hepatic fibrosis, an integrated cellular response to tissue injury. J. Biol. Chem..

[148] Geerts A. (2001). History, heterogeneity, developmental biology, and functions of quiescent hepatic stellate cells. Semin. Liver Dis..

[149] Mallat A., Lotersztajn S. (2013). Cellular mechanisms of tissue fibrosis. 5. Novel insights into liver fibrosis. Am. J. Physiol. Cell. Physiol..

[150] Yin C.H., Evason K.J., Asahina K., Stainier D. (2013). Hepatic stellate cells in liver development, regeneration, and cancer. J. Clin. Investig..

[151] Forbes S.J., Parola N. (2011). Liver fibrogenic cells. Best Pract. Res. Clin. Gastroenterol..

[152] Taura K., Miura K., Iwaisako K., Osterreicher C.H., Kodama Y., Penz-Osterreicher M., Brenner D.A. (2010). Hepatocytes do not undergo epithelial-mesenchymal transition in liver fibrosis in mice. Hepatology.

[153] Liu Y., Liu H., Meyer C., Li J., Nadalin S., Königsrainer A., Weng H., Dooley S., Dijke P. (2013). TGF-β mediated connective tissue growth factor (CTGF) expression in hepatic stellate cells requires Stat3 activation. J. Biol. Chem..

[154] Yanagitani A., Yamada S., Yasui S., Shimomura T., Murai R., Murawaki Y., Hashiguchi K., Kanbe T., Saeki T., Ichiba M. (2004). Retinoic acid receptor a dominant negative form causes steatohepatitis and liver tumors in transgenic mice. Hepatology.

[155] Shirakami Y., Lee S.A., Clugston R.D., Blaner W.S. (2012). Hepatic metabolism of retinoids and disease associations. Biochim. Biophys. Acta.

[156] Esteban-Pretel G., Marín M.P., Cabezuelo F., Moreno V., Renau-Piqueras J., Timoneda J., Barber T. (2010). Vitamin A deficiency increases protein catabolism and induces urea cycle enzymes in rats. J. Nutr..

[157] Tsukamoto H., She H., Hazra S., Cheng J., Wang J. (2008). Fat paradox of steatohepatitis. J. Gastroenterol. Hepatol..

[158] Kim H.Y., Wolf G. (1987). Vitamin A deficiency alters genomic expression for fibronectin in liver and hepatocytes. J. Biol. Chem..

[159] Seifert W.F., Bosma A., Brouwer A., Hendriks H.F., Roholl P.J., van Leeuwen R.E., van Thiel-de Ruiter G.C., Seifert-Bock I., Knook D.L. (1994). Vitamin A deficiency potentiates carbon tetrachloride-induced liver fibrosis in rats. Hepatology.

[160] Wang L., Tankersley L.R., Tang M., Potter J.J., Mezey E. (2002). Regulation of the murine α2(I) collagen promoter by retinoic acid and retinoid X receptors. Arch. Biochem. Biophys..

[161] Meisler N.T., Parrelli J., Gendimenico G.J., Mezick J.A., Cutroneo K.R. (1997). All-trans retinoic acid inhibition of pro-α1(I) collagen gene expression in fetal rat skin fibroblasts: Identification of a retinoic acid response element in the pro- α1(I) collagen gene. J. Investig. Dermatol..

[162] Cohen A.J., Lassová L., Golden E.B., Niu Z., Adams S.L. (2006). Retinoids directly activate the collagen X promoter in prehypertrophic chondrocytes through a distal retinoic acid response element. J. Cell. Biochem..

[163] Pan L., Eckhoff C., Binckerhoff C.E. (1995). Suppression of collagenase gene expression by all-trans and 9-cis retinoic acid is ligand dependent and requires both RARs and RXRs. J. Cell. Biochem..

[164] Zaragozá R., Gimeno A., Miralles V.J., García-Trevijano E.R., Carmena R., García C., Mata M., Puertes I.R., Torres L., Viña J.R. (2007). Retinoids induce MMP-9 expression through RARα during mammary gland remodeling. Am. J. Physiol. Endocrinol. Metab..

[165] Lackey D.E., Hoag K.A. (2010). Vitamin A upregulates matrix metalloproteinase-9 activity by murine myeloid dendritic cells through a nonclassical transcriptional mechanism. J. Nutr..

[166] Glick A.B., McCune B.K., Abdulkarem N., Flanders K.C., Lumadue J.A., Smith J.M., Sporn M.B. (1991). Complex regulation of TGFβ expression by retinoic acid in the vitamin A-deficient rat. Development.

[167] Mercier T., Gaillard-Sanchez I., Martel P., Heberden C. (1996). TGF-β receptors are diminished after retinoid exposure in rat liver epithelial cells. J. Cell. Biochem..

[168] Morath C.V., Dechow C., Lehrke I., Haxsen V., Waldherr R., Floege J., Ritz E., Wagner J. (2001). Effetcs of retinoids on the TGF-β system and extracellular matrix in experimental glomerulonephritis. J. Am. Soc. Nephrol..

[169] Verrecchia F., Chu M-L., Mauviel A. (2001). Identification of novel TGF-β/Smad gene targets in dermal fibroblast using a combined cDNA microarray/promoter transactivation approach. J. Biol. Chem..

[170] Zeisberg L., Kalluri R. (2004). The role of epithelial-to-mesenchymal transition in renal fibrosis. J. Mol. Med..

[171] Willis B.C., duBois R.M., Borok Z. (2006). Epithelial origin of myofibroblasts during fibrosis in the lung. Proc. Am. Thorac. Soc..

[172] Lamouille S., Xu J., Derynck R. (2014). Molecular mechanisms of epithelial-mesenchymal transition. Nat. Rev. Mol. Cell Biol..

[173] Gatica L.V., Oliveros L.B., Pérez Díaz M.F., Domínguez N.S., Fornes M.W., Gimenez M.S. (2012). Implication of vitamin A deficiency on vascular injury related to inflammation and oxidative stress. Effects on the ultrastructure of rat aorta. Eur. J. Nutr..

[174] Shimada J., Suzuki Y., Kim S.J., Wang P.C., Matsumura M., Kojima S. (2001). Transactivation via 632 RAR/RXR-Sp1 interaction: Characterization of binding between Sp1 and GC box 633 motif. Mol. Endocrinol..

[175] Schmidt C., Fischer G., Kadner H., Genersch E., Kühn K., Pöschl E. (1993). Differential effects of DNA-binding proteins on bidirectional transcription from the common promoter region of human collagen type IV genes COL4A1 and COL4A2. Biochim. Biophys. Acta.

[176] Tsang K.J., Crowe D.L. (2001). Retinoic acid and extracellular matrix inhibition of matrix metalloproteinase 9 expression is mediated by mitogen activated protein kinase pathway. Int. J. Oncol..

[177] Barber T., Borrás E., Torres L., García C., Cabezuelo F., Lloret A., Pallardó F.V., Viña J.R. (2000). Vitamin A deficiency causes oxidative damage to liver mitochondria in rats. Free Radic. Biol. Med..

[178] Iglesias de la Cruz M.C., Ruiz-Torres P., Alcammí J., Díez-Marqués L., Ortega-Velazquez R., Chen S., Rodríguez-Puyol M., Ziyadeh F.N., Rodríguez-Puyol D. (2001). Hydrogen peroxide increases extracelular matrix mRNA through TGF-β in human mesangial cells. Kidney Int..

[179] Liu R.M., Gaston Pravia K.A. (2010). Oxidative stress and glutathione in TGF-β-mediated fibrogenesis. Free Radic. Biol. Med..

[180] Rhyu D.Y., Park J., Sharma B.R., Ha H. (2012). Role of reactive oxygen species in transforming groth factor-beta-1-induced extracellular matrix accumulation in renal tubular epithelial cells. Transplant. Proc..

[181] Samarakoon R., Overstreet J.M., Higgins P.J. (2013). TGF-β signaling in tissue fibrosis: Redox controls, target genes and therapeutic opportunities. Cell. Signal..

[182] Aoujehane L., Pissaia A., Scatton O., Podevin P., Massault P.-P., Chouzenoux S., Soubrane O., Calmus Y., Conti F. (2008). Interleukine-4 induces the activation and collagen production of cultured human intrahepatic fibroblast via the STAT-6 pathway. Lab. Investig..

[183] Mir S.A., Chatterjee A., Mitra A., Pathak K., Mahata S., Sarkar S. (2012). Inhibition of signal transducer and activator of transcription 3 (STAT3) attenuates interleukin-6 (IL-6)-induced collagen synthesis and resultant hypertrophy in rat heart. J. Biol. Chem..

[184] Matsui F., Rhee A., Hile K.L., Zhang H., Meldrum K.K. (2013). IL-18 induces profibrotic renal tubular cell injury via STAT3 activation. Am. J. Physiol. Renal Physiol..

[185] Dai B., Cui M., Zhu M., Su W.L., Qiu M.C., Zhang H. (2013). STAT 1/3 and ERK1/2 synergistically regulate cardiac fibrosis induced by high glucose. Cell. Physiol. Biochem..

[186] Robledo T., Arriaga-Pizano L., Lopez-Pérez M., Pérez Salazar E. (2005). Type IV collagen induces STAT5 activation in MCF7 human breast cancer cells. Matrix Biol..

[187] Cortes-Reynosa P., Robledo T., Macias-Silva M., Vincent Wu S., Perez Salazar E. (2008). Src kinase regulates metalloproteinase-9 secretion induced by type IV collagen in MCF-7 human breast cancer cells. Matrix Biol..

[188] Shintani Y., Maeda M., Chaika N., Johnson K.R., Wheelock M.J. (2008). Collagen I promotes epithelial-to-mesenchimal transition in lung cancer cells via transforming growth factor-β signaling. Am. J. Respir. Cell. Mol. Biol..

[189] Zhou T.B., Drummen G.P., Qin Y.H. (2012). The controversial role of retinoic acid in fibrotic diseases: Analysis of involved signaling pathways. Int. J. Mol. Sci..

[190] Massaro D., Massaro G.D. (1997). Retinoic acid treatment abrogates elastase-induced pulmonary emphysema in rats. Nat. Med..

[191] Wagner J., Dechow C., Morath C., Lehrke I., Amann K., Waldherr R., Floege J., Ritz E. (2000). Retinoic acid reduces glomerular injury in a rat model of glomerular damage. J. Am. Soc. Nephrol..

[192] Veness-Meehan K.A., Pierce R.A., Moats-Staats B.M., Stiles A.D. (2002). Retinoic acid attenuates O_2_-induced inhibition of lung septation. Am. J. Physiol. Lung Cell. Mol. Physiol..

[193] Massaro D., Massaro G.D. (2006). Toward therapeutic pulmonary alveolar regeneration in humans. Proc. Am. Thorac. Soc..

[194] James M.L., Ross A.C., Nicola T., Steele C., Ambalavanan N. (2013). VARA attenuates hyperoxia-induced impaired alveolar development and lung function in newborn mice. Am. J. Physiol. Lung Cell. Mol. Physiol..

[195] Takahashi N., Takasu S. (2011). A close relationship between type 1 diabetes and vitamin A-deficiency and matrix metalloproteinase and hyaluronidase activities in skin tissues. Exp. Dermatol..

[196] Oseto S., Moriyama T., Kawda N., Nagatoya K., Takeji M., Ando A., Yamamoto T., Imai E., Hori M. (2003). Therapeutics effects of all-trans retinoic acid on rats with anti-GBM antibody glomerulonephritis. Kidney Int..

[197] Davis B.H., Kramer R.T., Davidson N.O. (1990). Retinoic acid modulates rat Ito cell proliferation, collagen, and transforming growth factor beta production. J. Clin. Investig..

[198] Ye Y., Dan Z. (2010). All-trans retinoic acid diminishes collagen production in a hepatic stellate cell line via suppression of active protein-1 and c-Jun *N*-terminal kinase signal. J. Huazhong Univ. Sci. Technol. Med. Sci..

